# A Motion-Based Feature for Event-Based Pattern Recognition

**DOI:** 10.3389/fnins.2016.00594

**Published:** 2017-01-04

**Authors:** Xavier Clady, Jean-Matthieu Maro, Sébastien Barré, Ryad B. Benosman

**Affiliations:** Centre National de la Recherche Scientifique, Institut National de la Santé Et de la Recherche Médicale, Institut de la Vision, Sorbonne Universités, UPMC University Paris 06Paris, France

**Keywords:** neuromorphic sensor, event-driven vision, pattern recognition, motion-based feature, speed-tuned integration time, histogram of oriented optical flow, corner detection, gesture recognition

## Abstract

This paper introduces an event-based luminance-free feature from the output of asynchronous event-based neuromorphic retinas. The feature consists in mapping the distribution of the optical flow along the contours of the moving objects in the visual scene into a matrix. Asynchronous event-based neuromorphic retinas are composed of autonomous pixels, each of them asynchronously generating “spiking” events that encode relative changes in pixels' illumination at high temporal resolutions. The optical flow is computed at each event, and is integrated locally or globally in a speed and direction coordinate frame based grid, using speed-tuned temporal kernels. The latter ensures that the resulting feature equitably represents the distribution of the normal motion along the current moving edges, whatever their respective dynamics. The usefulness and the generality of the proposed feature are demonstrated in pattern recognition applications: local corner detection and global gesture recognition.

## 1. Introduction

In computer vision, a feature is a more or less compact representation of visual information that is relevant to solve a task related to a given application (see Laptev, 2005; Mikolajczyk and Schmid, 2005; Mokhtarian and Mohanna, 2006; Moreels and Perona, 2007; Gil et al., 2010; Dickscheid et al., 2011; Gauglitz et al., 2011). Building a feature consists in encoding information contained in the visual scene (global approach) or in a neighborhood of a point (local approach). It can represent static information (e.g., shape of an object, contour, etc.), dynamic information (e.g., speed and direction at the point, dynamic deformations, etc.) or both simultaneously.

**Figure 1:**
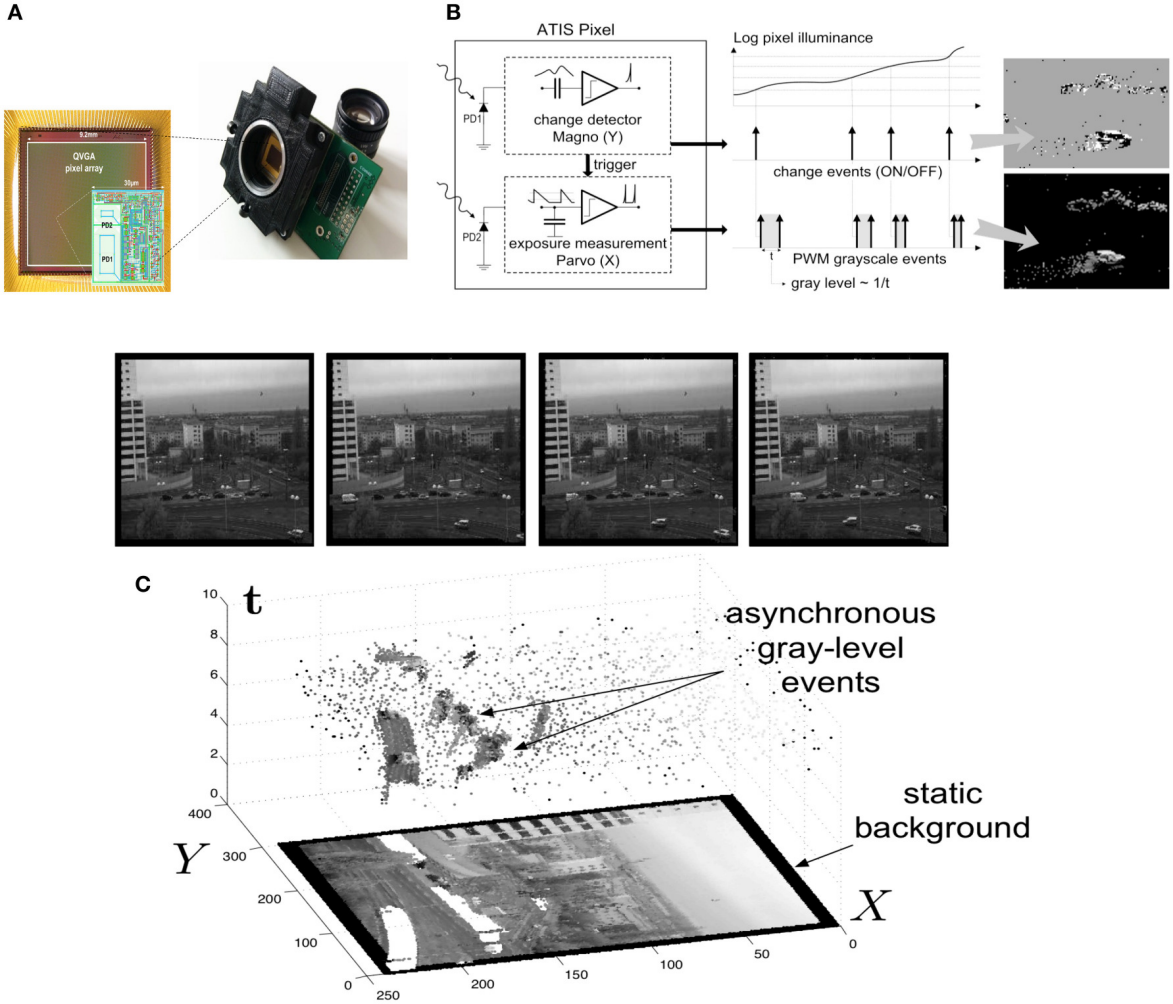
**ATIS, Asynchronous Time-based Image Sensor: (A)** The ATIS and its pixel array, made of 304 × 240 pixels (QVGA). PD1 is the change detector, PD2 is the grayscale measurement unit. **(B)** When a contrast change occurs in the visual scene, the ATIS outputs a change event (ON or OFF) and a grayscale event. **(C)** The spatio-temporal space of imaging events: static objects and scene background are acquired first. Then, dynamic objects trigger pixel-individual, asynchronous gray-level events after each change. Frames are absent from this acquisition process. Samples of generated images from the presented spatio-temporal space are shown in the upper part of the figure.

**Figure 2:**
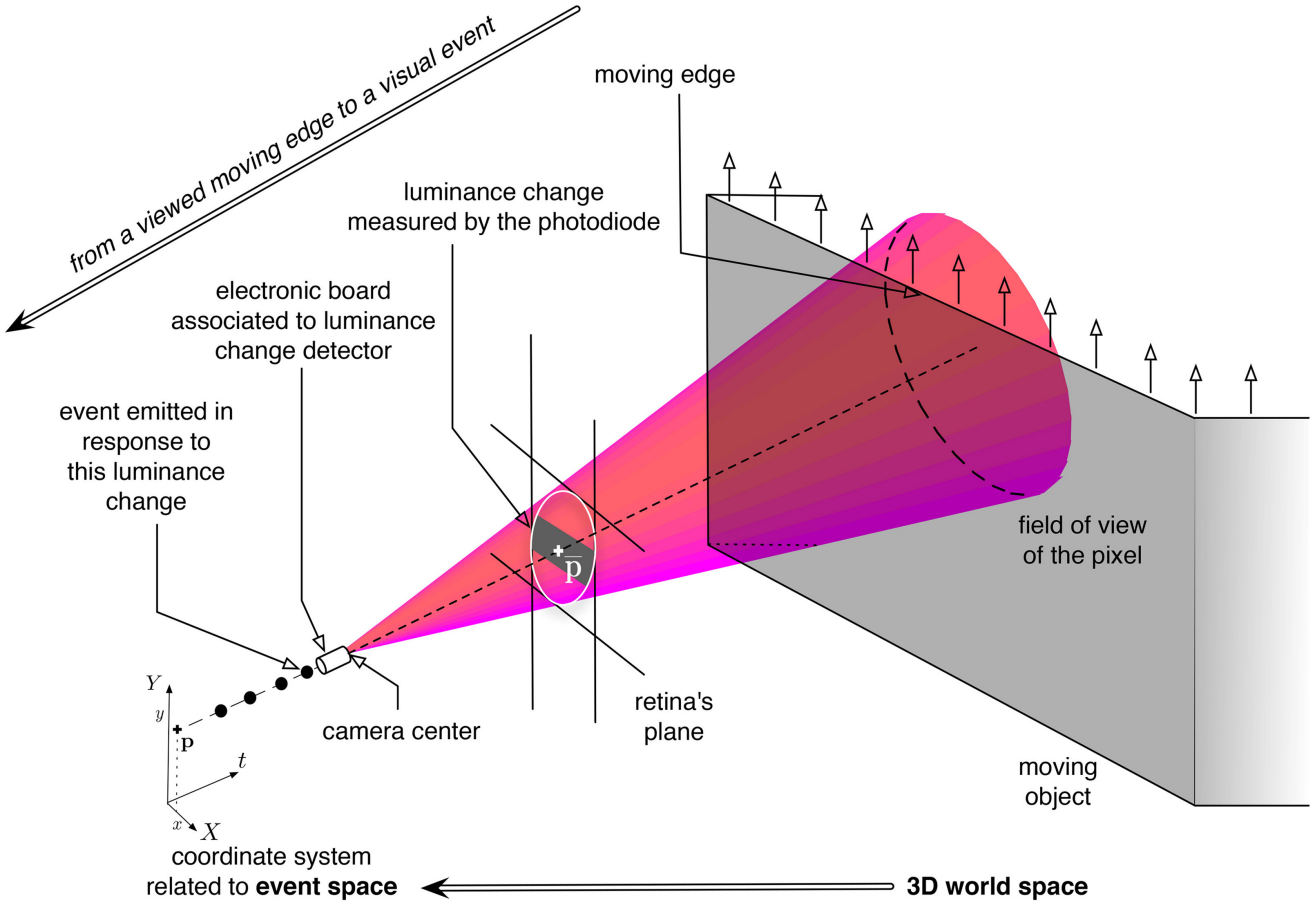
**Illustration of the luminance change measured by a neuromorphic pixel, modeled as a cone-pixel (Debaecker et al., 2010), viewing an moving edge**. **p** are the coordinates of the center of the pixel, in the coordinate system related to the retina's plane. The emitted event in response to this luminance change is represented as a black dot in the coordinate system *XYt* related to the event space (in the lower-left part of the figure).

In this article, we propose a motion-based feature computed on visual information provided by asynchronous image sensors known as neuromorphic retinas (see Delbrück et al., 2010; Posch, 2015). These cameras provide visual information as asynchronous event-based streams while conventional cameras output it as synchronous frame-based streams. The ATIS (“Asynchronous Time-based Image Sensor,” Posch et al., 2010; Posch, 2015), one of the neuromorphic visual sensors used in this work, is a time-domain encoding image sensor with QVGA resolution. It contains an array of fully autonomous pixels that combine an illuminance change detector circuit, associated to the PD1 photodiode, see Figure 1A and a conditional exposure measurement block, associated to the PD2 photodiode. The change detector individually and asynchronously initiates the measurement of an exposure/gray scale value only if a brightness change of a certain magnitude has been detected in the field-of-view of the respective pixel, as shown in the functional diagram of the ATIS pixel in Figures 1B, 2. The exposure measurement circuit encodes the absolute instantaneous pixel illuminance into the timing of asynchronous event pulses, more precisely into inter-event intervals. The DVS (“Dynamic Visual Sensor,” Lichtsteiner et al., 2008; Serrano-Gotarredona and Linares-Barranco, 2013), another neuromorphic camera used in this work, works in a similar manner but only the illuminance change detector is implemented and retina's spatial resolution is limited to 128 × 128*pixels*.

Despite the recent introduction of neuromorphic cameras, numerous applications have already emerged in robotics (see Censi et al., 2013; Delbrück and Lang, 2013; Lagorce et al., 2013; Clady et al., 2014; Ni et al., 2014; Milde et al., 2015), shape tracking (see Drazen et al., 2011; Ni et al., 2015; Valeiras et al., 2015), stereovision (cf. Rogister et al., 2012; Carneiro et al., 2013; Camuñas-Mesa et al., 2014; Firouzi and Conradt, 2015), corner detection (Clady et al., 2015), or shape recognition (see Pérez-Carrasco et al., 2013; Akolkar et al., 2015; Orchard et al., 2015a,b; Lee et al., 2016). This strong interest in such a sensor is essentially due to its ability to provide visual information as a high temporal resolution, luminance-free, and non-redundant stream. This makes it a fitting for high-speed applications [e.g., gesture recognition as in Lee et al. (2014), high-speed object tracking as in Lagorce et al. (2014), Mueggler et al. (2015a)].

The proposed feature consists in mapping the distribution of the optical flow along the contours of the objects in the visual scene into a matrix (see Section 2). It can be computed locally or more globally according to the targeted applications. Indeed, in the experimental evaluations, we propose to demonstrate its usefulness and generality in various applications. It is used to locally detect corners (see Section 3) or to summarize global motion observed in a scene in order to recognize actions, here hand gestures for an application in human-machine interaction (see Section 4).

## 2. Motion-based feature

Visual event streams are generated asynchronously at a high temporal resolution, essentially by moving edges. They are thus especially suitable for visual motion flow or optical flow (OF) computation (Benosman et al., 2014; Orchard and Etienne-Cummings, 2014; Brosch et al., 2015) along contours of objects. In the following sections, methods and mechanisms are proposed to estimate normal motion flows computed around events and to map them into a matrix in order to incrementally estimate scene motion distribution (locally or globally). This matrix will be considered as a feature. Its computation requires only the visual events provided by the change detectors of the retina (associated to photodiodes PD1 in Figure 1A), that can be defined as four components vectors:

(1) $$\begin{array}{l} \text{e}={\left(\text{p},t,pol\right)}^{T}, \end{array}$$

where **p** = (*x, y*)^*T*^ is the spatial coordinate of each event, *t*, its timestamp and *pol* ∈ {−1, 1} is the polarity, which is equal to −1/1 when the measured luminance decrease/increase is significant enough (see upper part of Figure 1B).

### 2.1. Extracting normal visual motion

We use the event-based OF computation method proposed in Benosman et al. (2014) which is known for its robustness and its algorithmic efficiency (see Clady et al., 2014, 2015; Mueggler et al., 2015b). More bio-inspired event-based OF computation methods such as Brosch et al. (2015) and Orchard and Etienne-Cummings (2014) can be used but they are computationally more expensive.

A function Σ_*e*_ that maps to each **p** the time *t* is defined locally:

$$\Sigma_{e}:\begin{array}{c} N^{2}\to R\\ \text{p}\mapsto t \end{array}$$

Applying the inverse function theorem of calculus, the vector ∇Σ_*e*_ measures the rate and the direction of change of time with respect to space: it is the normal optical flow, noted $\text{v}={\left(v_{x},v_{y}\right)}^{T}$, such as:

$$\nabla \Sigma_{e}={\left(\frac{1}{v_{x}},\frac{1}{v_{y}}\right)}^{⊤}$$

This equation could be defined assuming that the surface described by the visual events (generated by a moving edge) in the space-time reference frame (*XYt*)^*T*^ is continuous. This assumption is validated through a regularization process proposed in order to locally estimate this surface as a spatiotemporal plane (fitted directly on the local event stream). In this work the implementation proposed in Clady et al. (2015) has been chosen because it proposes mechanisms to automatically adapt the temporal dimension of the local neighborhood to the edge's dynamics, and to reject estimations of optical flow probably wrong and due to noise. This algorithm allows us to consider a function that associates for each valid visual event e∈E, a so-called visual motion event, noted **v**_**e**_, such as:

(2) $$\begin{array}{l} \begin{array}{c} E\to V\\ \text{e}={\left(\text{p},t,pol\right)}^{T}\mapsto {\text{v}}_{\text{e}}={\left(\text{p},t,v,\theta \right)}^{T} \end{array} \end{array}$$

where (*v*, θ)^*T*^ corresponds to the intensity (i.e., speed) and the direction of the normal visual flow.

Remark 1. *Note that the polarity of visual events is not conserved by the function (Equation 2). Indeed, in the applications proposed in this article, it is not useful to “memorize” if the visual flow has been computed on a positive or negative event stream. If required, the feature can be augmented in order to distinguish the distribution along “positive contours” from the one along “negative contours*.”

### 2.2. Computing and updating the feature

As we said, the feature corresponds to the estimated distribution of the optical flow along the (local or global) contours in the visual scene. This distribution is evaluated on a grid-based sampling in the polar reference frame of the visual flow, such as it is subdivided into an interval set ${\left\{\bar{{\text{v}}^{l}}\right\}}_{l}={\left\{{\left(\bar{\theta^{l}},\bar{v^{l}}\right)}^{T}\right\}}_{l}$ where $\bar{\theta^{l}}$ is an angle based interval and $\bar{v^{l}}$ is an intensity based interval. Such a discretization of the velocity subspace is consistent with biologic observations about orientation (cf. Hubel and Wiesel, 1962, 1968) and speed (cf. Priebe et al., 2006) selectivity in V1 cells and human psychophysical experiments about speed discrimination as in Orban et al. (1984) and Kime et al. (2014, 2016). Here, we parametrize the grid sampling mostly according to these biologic observations and human psychophysical experiments. However, its ranges and precisions could be set in relation with targeted tasks, optimizing them according to given performance criteria. We define the centers ${\left\{\theta^{l}\right\}}_{l}$ of the angle intervals such as: $\theta^{l}\in \left[0,\ldots ,2\pi \frac{i}{N_{\theta }},\ldots ,2\pi \frac{N_{\theta -1}}{N_{\theta }}\right]$, with *i* ∈ [0, *N*_θ_ − 1]; $\frac{2\pi }{N_{\theta }}$ is the length of the interval and thus the angular precision of the grid. With *N*_θ_ = 36, we barely reach the precision (~10°) observed for V1 simple cells (see Hubel and Wiesel, 1962, 1968). For the velocity intensity, we propose a non-regular speed-based sampling, where {*v*^*l*^} are the centers of the speed based intervals on a logarithmic scale. The sampling is then operated such that $v^{l}\in \left[v_{min},\ldots ,v_{min}\gamma^{i},\ldots ,v_{min}\gamma^{N_{v}-1}\right]$, with *i* ∈ [0, *N*_*v*_ − 1] and γ = 1 + ϵ_*v*_ (ϵ_*v*_ > 0). This discretization strategy ensures an *a priori* constant relative precision in speed estimation: $\frac{\Delta v^{l}}{v^{l}}\approx \epsilon_{v}$. Setting ϵ_*v*_ to 0.1 will barely correspond to the relative speed-discrimination threshold (10%) observed in human psychophysical experiments (see Orban et al., 1984; Kime et al., 2014, 2016). *v*_*min*_ has been fixed to 1*pixel*.*s*^−1^ and *N*_*v*_ to 73 in order that $v_{max}=v_{min}\gamma^{N_{v}-1}$ is close to 1000*pixels*.*s*^−1^, i.e., inversely close to the temporal precision of the visual events, estimated over 1 ms (cf. Akolkar et al., 2015). Motions with intensities less than *v*_*min*_ are then discarded: they are assumed as belonging to static or faraway objects in the background visual scene. Motions with intensities higher than *v*_*max*_ are also discarded because noise associated to their computation can *a priori* be considered as too high.

Finally, the feature, noted F∈F, is defined as a matrix corresponding to this grid, and associated to a spatiotemporal point (**p**, *t*)^*T*^ of the retina (or to the entire visual scene for a global approach), and computed as:

(3) $$\begin{array}{l} \begin{array}{c} \;V\to F\\ {\left\{{\text{v}}_{j}\right\}}_{j=1,\ldots ,N}\mapsto {\text{F}}_{\text{p},t}\left(v^{l},\theta^{l}\right)=\underset{j}{\sum }w_{v}\left(v_{j}-v^{l},\theta_{j}-\theta^{l}\right)w_{s}\left(\text{p}-{\text{p}}_{j}\right)\\ \;w_{t}^{l}\left(t-t_{j}\right) \end{array} \end{array}$$

where:

- *w*_*t*_ is a temporal exponentially decay function (or kernel), inspired by the synchrony measure of spike trains proposed in van Rossum (2001), such that:
  (4) $$\begin{array}{l} w_{t}^{l}\left(t-t_{j}\right)=H\left(t-t_{j}\right)\exp\left(-\alpha v^{l}\left(t-t_{j}\right)\right) \end{array}$$
  where *H*(·) is the Heaviside step function and α parametrizes the global decreasing dynamic. In our experiments (see Sections 3 and 4), we fixed α to 0.8, i.e., close to 1 in order to mostly take into account the current edges while slightly smoothing them in order to make **F** less sensitive to both noise and missing data. This kernel gives indeed more weight (or a higher probability value) to events generated by current edges, i.e., the events with timings close to *t*, while also respecting an isoprobabilistic representation of the edges whatever their dynamics, as we will discuss below (see Section 2.3). Of course, other temporal kernels [Gaussian-based in Schreiber et al. (2003),…] can be envisioned, but this one has the advantage of being causal and of leading to an incremental computation of the feature (see Equation 7).
- *w*_*s*_ is a spatial bivariate function, which can be defined as:
  1. in a global approach, *w*_*s*_(**p** − **p**_*j*_) = 1, which gives an equitable representation to the edges whatever their spatial locations, or
  2. in a local approach:
    (5) $$\begin{array}{l} w_{s}\left(\text{p}-{\text{p}}_{j}\right)=\frac{1}{2\pi \sigma_{s}^{2}}\exp\left(-\frac{||\text{p}-{\text{p}}_{j}|{|}^{2}}{2\sigma_{s}^{2}}\right), \end{array}$$
    where σ_*s*_ implicitly parametrizes the spatial scale of a region of interest or neighborhood around the spatial location **p**; **F**_**p**,*t*_ then represents the local distribution of the normal velocities around the spatiotemporal location (**p**, *t*)^*T*^;
- *w*_*v*_ is the multiplication of two univariate Gaussian-like functions used to take into account potential imprecisions in the computation of the optical flow, defined as:
  (6) $$\begin{array}{l} w_{v}\left(v_{j}-v^{l},\theta_{j}-\theta^{l}\right)=\exp\left(-\frac{{\left(v_{j}-v^{l}\right)}^{2}}{V^{2}}\right)\exp\left(-\frac{{\left(\theta_{j}-\theta^{l}\right)}^{2}}{\Theta^{2}}\right) \end{array}$$
  with $V^{2}=v_{j}v^{l}$ in order to consider a relative speed imprecision, and Θ set to 20°. So, even if an estimated motion belongs to a wrong interval because of noise, it will still contribute to the right element of the matrix, probably close.

As we said previously, the feature can be incrementally updated at each occurring visual motion event **v**_*i*_, considering that ${\text{F}}_{\text{p},0}\left(v^{l},\theta^{l}\right)=\frac{1}{{\text{N}}_{\theta }{\text{N}}_{v}}$ for all (*v*^*l*^, θ^*l*^)^*T*^ (in order to consider, at time *t* = 0, an uniform distribution for the considered velocity-space), such as:

(7) $$\begin{array}{l} F_{p,t_{i}}(v^{l},\theta^{l})=F_{p,t_{i-1}}(v^{l},\theta^{l})\exp\left(-\alpha v^{l}(t_{i}-t_{i-1})\right)\\ \;+\;w_{s}(p-p_{i})w_{v}(v_{i}-v^{l},\theta_{i}-\theta^{l}) \end{array}$$

Remark 2. *The feature works like a voting matrix, i.e., each visual motion event votes for the speed and direction interval it belongs (and its neighboring intervals through the weighting kernel *w*_*v*_, Equation 6). More visual events there are, more robust the feature will be. Conversely, the feature will be more sensitive to noise in low light or low contrast situations*.

*In addition the feature* **F** *can be related to a probabilistic distribution while normalizing it to sum up to 1, i.e., to divide it with $\underset{l}{\sum }\text{F}\left(v^{l},\theta^{l}\right)$*.

*In the global approach*, **F**_**p**,*t*_ *is independent of* **p**; *it can then be noted* **F**_*t*_*. Note that the feature is noted* **F** *(without sub-index) in this article when the application context (local or global approach) is not relevant or obvious*.

### 2.3. Speed-tuned vs. fixed decreasing strategies

Another important point to highlight is that the temporal decreasing function *w*_*t*_ (Equation 4) is related to the speed *v*^*l*^. Indeed, $\tau^{l}=\frac{1}{v^{l}}$ is the time during which an edge travels through a pixel or in other words, the estimated lifetime of its observation at a given location **p**, as already remarked in Clady et al. (2015) and Mueggler et al. (2015b). Including it as decay factor in the temporal kernel (Equations 4 and 7) provides a more isoprobabilistic representation of the moving edges in **F**, i.e., depending only of their contrasts whatever their respective dynamics.

**Figure 3:**
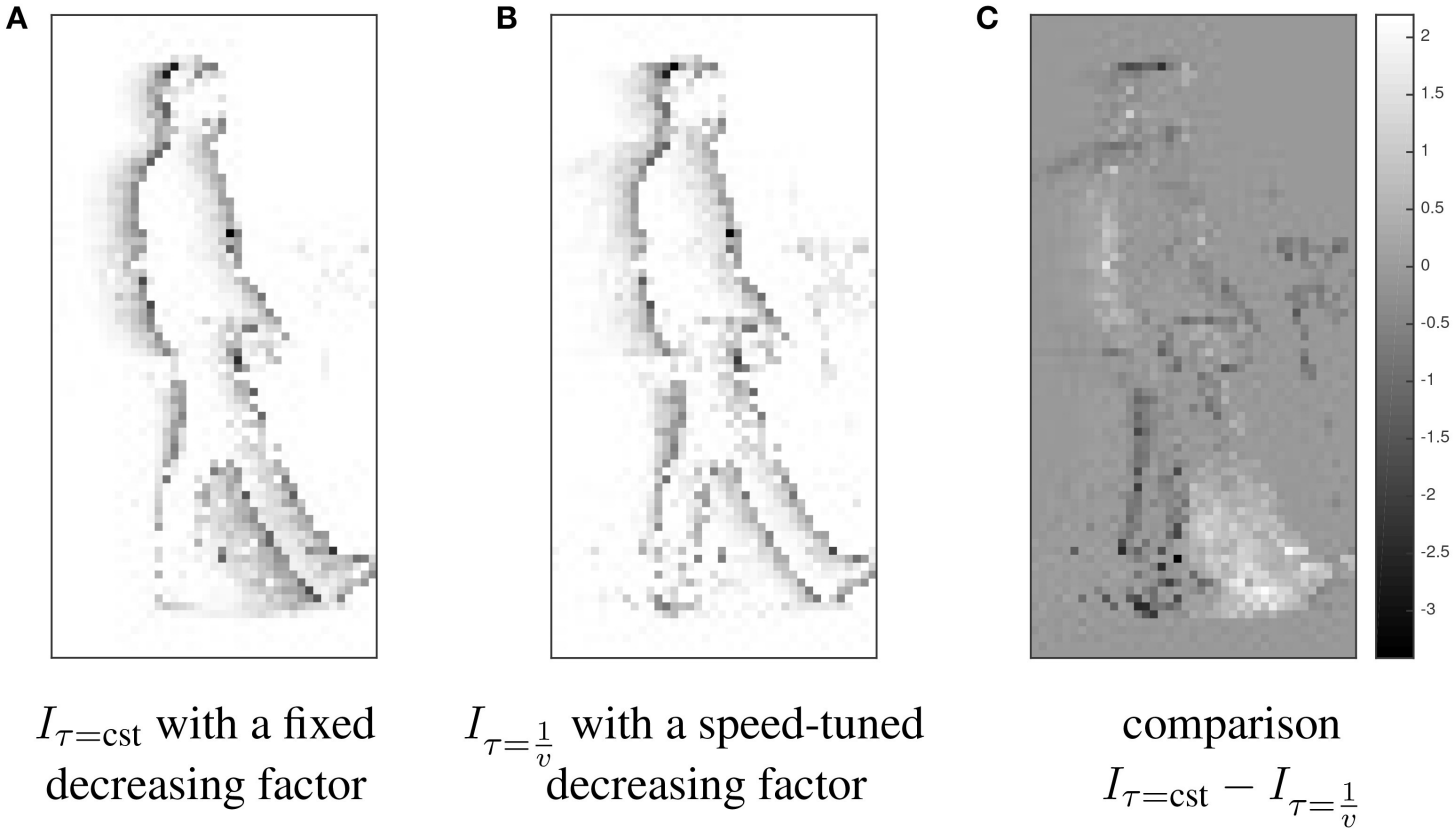
**Illustration of different strategies for the exponential decay function; comparison between synchrony images ***I*** built applying exponential temporal kernels with a constant decreasing factor (A)** and with a speed-tuned decreasing factor **(B)** to an event stream (acquired from a visual scene containing a walking person). The comparison $I_{\tau =\text{cst}}-I_{\tau =\frac{1}{v}}$ **(C)** of both images shows that the second strategy provides a more isoprobabilistic representation of the edges (taking into account the observation lifetime of the moving edges as in Clady et al., 2015; Mueggler et al., 2015b) than the first one; the high-velocity edges (resulting from the moving and forward leg) are over-represented and the low-velocity edges (resulting from the backward leg) are under-represented in the left synchrony image. **(A)** *I*_τ = cst_ with a fixed decreasing factor. **(B)** $I_{\tau =\frac{1}{v}}$ with a speed-tuned decreasing factor. **(C)** comparison $I_{\tau =\text{cst}}-I_{\tau =\frac{1}{v}}$.

In order to concretely illustrate this point, Figure 3 represents two synchrony images *I* built integrating a visual event stream and with two different strategies for decay factor τ (related to the speed or not), such as, for each occurring visual event **e**_*i*_, $I(p,t_{i})=I(p,t_{i-1})\exp\left(-\frac{t_{i}-t_{i-1}}{\tau }\right)+\delta (||p-p_{i}||)$ where δ(·) is the Dirac function. The left image (Figure 3A) results from this equation with a constant τ = cst (whatever the dynamics of the edges), and the middle image (Figure 3B) with a speed-tuned $\tau =\frac{1}{v}$. As shown in the right image (Figure 3C), which is the subtraction of both previous images without a speed-tuned factor the high-velocity edges (resulting from the moving and forward leg) are over-represented and the low-velocity edges (resulting from the backward leg) are under-represented in the corresponding synchrony image (Figure 3A). The moving edges are more equitably represented in the second synchrony image (Figure 3B) with a speed-tuned temporal kernel and, by extension, in feature **F**. Results in Section 3.2 show this equitable representation is very important to obtain accurate results.

The proposed strategy is also consistent with biological observations. Indeed Bair and Movshon (2004) showed that the effective integration time of the computations in direction-selective cells changes with stimulus speed; the integration time for slow motions is longer than that for fast motions. This is modeled in Equation (4) as a decay factor inversely proportional to the speed intensity.

The organization of the feature in a polar coordinate frame based grid, greatly facilitates its computation and its update. The representation of the visual motion information into speed and direction coordinates grants that each speed-tuned decay factor can be associated to an element of the grid, and not directly to the velocity associated to the occurring visual motion event. The latter indicates only which elements in the grid have to be incremented. A bio-inspired implementation can be envisioned where visual motion events are conveyed by selective lines (each line conveying only the motion events **v**_**e**_ included in its associated interval, ${\left(v,\theta \right)}^{T}\in {\left(\bar{v^{l}},\bar{\theta^{l}}\right)}^{T}$) from a neuron layer computing the optical flow to a leaky integrate-and-fire (LIF) neural layer (cf. Gerstner and Kistler, 2002), in which each neuron could be assimilated with an element of the feature; this selectivity of lines could result from the selectivity of neurons in the first neuron layer.

Indeed the following model (notations are inspired by Lee et al., 2016) can be used to update the membrane potential of a LIF neuron for a given input event (or spike):

(8) $$\begin{array}{l} V_{mp}\left(t_{i}\right)=V_{mp}\left(t_{i-1}\right)\exp\left(-\frac{t_{i}-t_{i-1}}{\tau_{mp}}\right)+w_{k}w_{dyn} \end{array}$$

where τ_*mp*_ is the membrane time constant, *w*_*k*_ is the synaptic weight of the k-th synapse (through which the input event or spike arrives) and *w*_*dyn*_ is a dynamic weight controlling a refractory period (see Gerstner and Kistler, 2002; Lee et al., 2016 for more details). This model is very similar to the incremental updating equation of our feature, Equation (7). The only things missing are the dynamic weight *w*_*dyn*_ and a firing threshold *V*_*th*_ in order to output approximatively the value of the corresponding feature's element as an event stream (or spike train), and then approximatively following a rate-coding model. Here, the refractory period should be set close to 0 (probably as a small fraction of the integration time $\tau^{l}=\frac{1}{v^{l}}$), in order to allow (quasi-)simultaneous visual events in the neighborhood (i.e., the events generate by the same contour moving across several pixels in the neighborhood) to contribute equitably to the neuron's potential, i.e., the value of the corresponding element of the feature.

For the local approach, a leaky integrate-and-fire neural layer has to be implemented for each pixel; this neural layer collects the visual motion events from the receptive field, Ω_**p**_*i*__ (defined as ∣∣ **p** − **p**_*i*_ ∣∣< 2σ_*s*_) defined by the corresponding bi-variate spatial kernel (Equation 5). This local computation is detailed in Algorithm 1. For the global approach, only one neural layer is required, collecting the visual motion events estimated over the entire retina.

**Table:** **Algorithm 1** Computation of the local feature.

1: **for all** pixel's location **p** ∈ Retina **do**
2: Set ${\text{F}}_{\text{p},0}\left(v^{l},\theta^{l}\right)=\frac{1}{{\text{N}}_{\theta }{\text{N}}_{v}}$ for all (*v*^*l*^, θ^*l*^)^*T*^
3: **end for**
4: **for all** event **e** = (**p**, *t, pol*)^*T*^ **do**
5: Compute the current optical flow ${\text{v}}_{\text{e}}={\left(\text{p},t,v,\theta \right)}^{T}$ (see Section 2.1).
6: **for all p**_*i*_ ∈ Ω_**p**_, where Ω_**p**_*i*__ is a spatial neighborhood such as ∣∣ **p** − **p**_*i*_ ∣∣ < 2σ_*s*_, **do**
7: Update **F**_**p**_*i*_,*t*_*i*__: ${\text{F}}_{{\text{p}}_{i},t}\left(v^{l},\theta^{l}\right)={\text{F}}_{{\text{p}}_{i},t_{i}}\left(v^{l},\theta^{l}\right)\exp\left(-\alpha v^{l}\left(t-t_{i}\right)\right)+w_{s}\left(\text{p}-{\text{p}}_{i}\right)w_{v}\left(v-v^{l},\theta -\theta^{l}\right)$,
where *t*_*i*_ is the timing of the previous update of **F**_**p**_*i*_, *t*_ (see Equation 7)
8: **end for**
9: **end for**
10: Output **F**_**p**,*t*_

**Figure 4:**
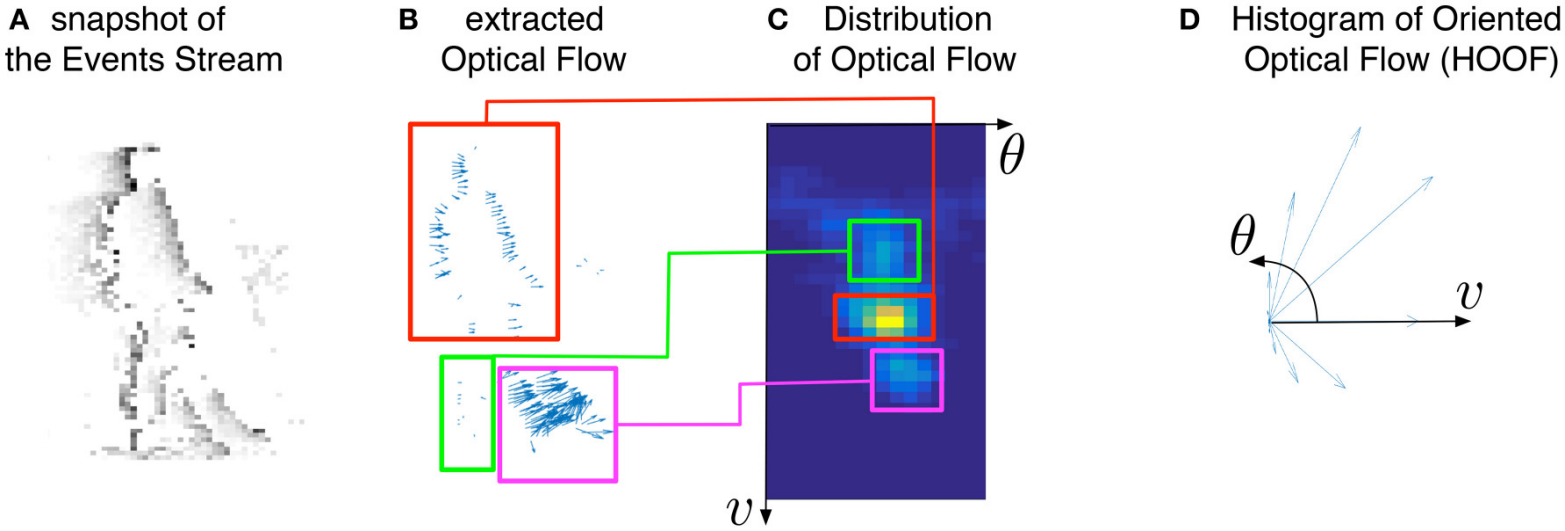
**Illustration of the global motion-based feature for event-based vision: from the stream of events (A)**, the optical flow **(B)** is extracted. The feature corresponds to the distribution of this optical flow **(C)** in a polar coordinate frame, and can be reduced into a more compact and scale-invariant representation, called Histogram of Oriented Optical Flow **(D)** (see Section 4.1). As we can see in **(B,C)**, the motions generated by the forward leg (magenta boxes), the backward leg (green boxes), and the rest of body (red boxes) corresponds to three distinct and representative modes in the proposed feature.

Finally, Figure 4 shows that the distribution of optical flow representation in the global approach (Figure 4C) summarizes the principal motions observed in the visual scene. This property will allow us to propose a machine learning based approach to recognize gestures in Section 4. In the next Section, we will demonstrate that the local version can be also used to detect particular interest points, i.e., corners.

Remark 3. *If the photodiode of the retina's pixel is not square as for the ATIS's one (see Posch et al., 2010 and Figure 1A), the frequency of a set of events emitted by a pixel will be not the same when a contour moves horizontally or vertically in the pixel's field of view (contour's speed and contrast are considered equal in both cases), because the contour travels the same surface of the photodiode during different time periods. In this case, keeping a decay factor invariant whatever the direction of the motion will introduce a bias, favoring one direction over another, in* **F**. *To avoid this bias, a cone-pixel with an ellipse-based basis (and not a disk-based basis as illustrated in Figure 2) can be implicitly considered in a correcting function α_θ_(·) introduced in Equations 4 and 7 (instead of the constant smoothing parameter α); it is depending on the direction θ^*l*^ of the visual motion and defined as*:

(9) $$\begin{array}{l} \alpha_{\theta }\left(\theta^{l}\right)=\alpha \sqrt{\frac{1}{1-e^{2}\cos{\left(\theta^{l}\right)}^{2}}} \end{array}$$

*where* α ∈ [0, 1] *and* $e=\sqrt{1-{\left(\frac{a}{b}\right)}^{2}}$ *is the eccentricity of the ellipse, with *a* and *b* the width and the length of the photodiode, respectively. The second term of this equation increases the decay factor in the direction of the principal axis of the ellipse, rebalancing the representation of the moving edges in* **F**.

## 3. Application to corner detection

In conventional frame-based vision, several techniques have been proposed that consist in determining points for which a measurement is locally optimal with respect to a criteria; in particular specific to corners. This measure can be computed by a cumulative process (Park et al., 2004), using a self-similarity measure (Moravec, 1980) derived from mathematical analysis [e.g., contour's local curvature (Mokhtarian and Suomela, 1998), relying on an eigenvalue decomposition of a second-moment matrix (Harris and Stephens, 1988)] or selected as the output from a machine learning process (Rosten and Drummond, 2006).

**Figure 5:**
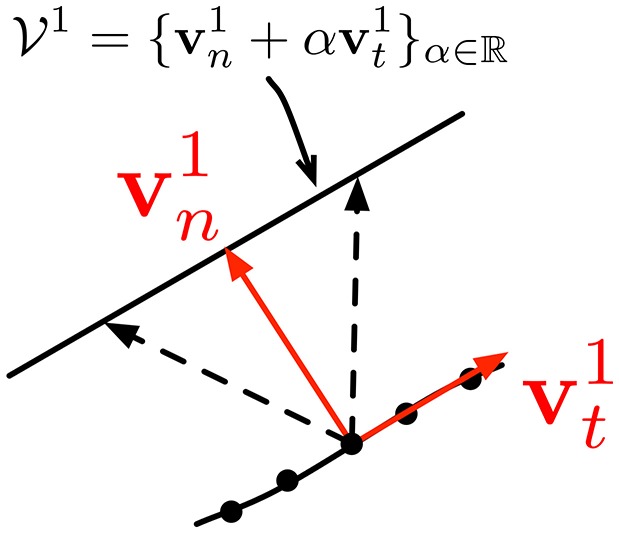
**The aperture problem allows estimating only the normal component ${\text{v}}_{1}^{n}$ of the velocity of events generated by an edge**. The tangential component ${\text{v}}_{t}^{1}$ is not recoverable. Any motion with the same component ${\text{v}}_{n}^{1}$ induces the same stimulus. These motions define the real plane subspace $V^{1}$. (extracted from Clady et al., 2015).

In asynchronous event-based vision, Clady et al. (2015) have proposed an algorithm based on the intersection of constraints principle (see Adelson and Movshon, 1982); which considers corners as locations where the aperture problem can be solved locally. Since cameras have a finite aperture size, motion estimation is possible only for directions orthogonal to edges. Figure 5 shows the ambiguity due to the finite aperture. This can be written as follows: if **v**_*n*_ is the normal component of the velocity vector to an edge at time *t* at a location **p**, then the real velocity vector is an element of the ℝ^2^ subspace spanned by the unit vector **v**_*t*_, tangent to the edge at **p**. This subspace is defined as $V^{1}=\left\{\text{v}={\text{v}}_{n}+\alpha {\text{v}}_{t}\right\}$ with α ∈ ℝ. For a regular edge point, α can usually not be estimated. When two moving crossed gratings are superimposed to produce a coherent moving pattern, the velocity can be unambiguously estimated.

**Figure 6:**
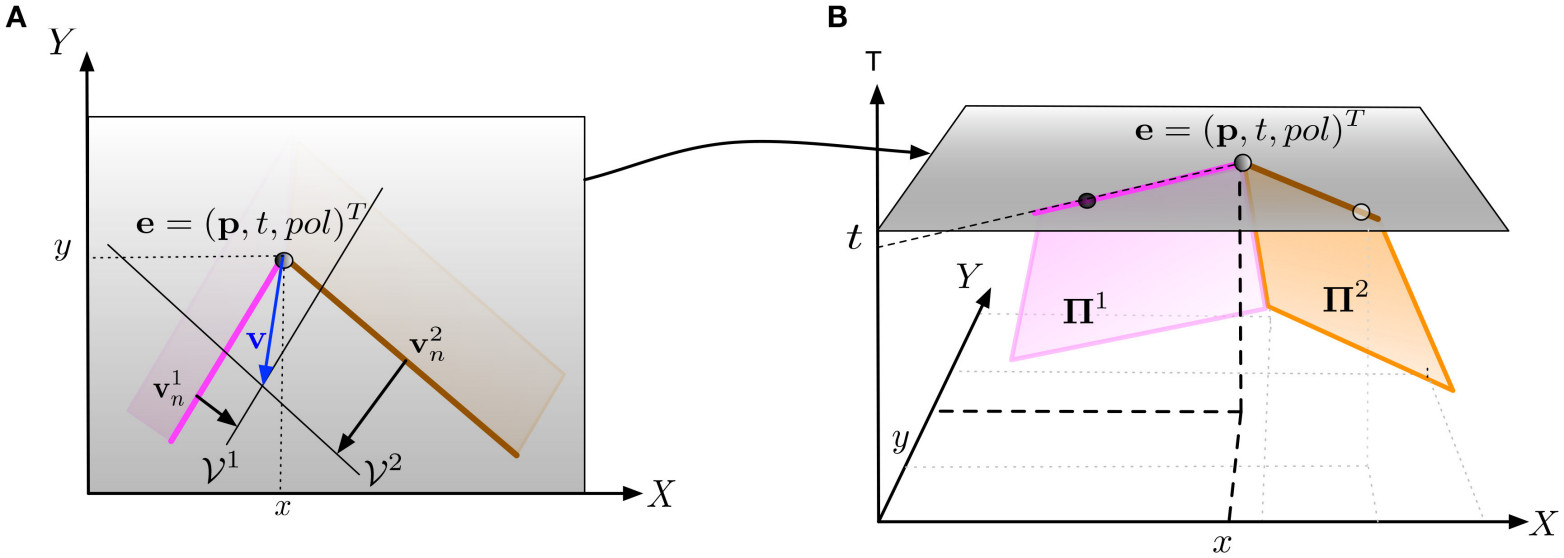
**(A)** An event *e* occurs at spatial location **p** at time *t* where two edges intersect. This configuration provides sufficient constraints to estimate the velocity **v** at **p** from the normal velocity vector ${\text{v}}_{n}^{1}$ and ${\text{v}}_{n}^{2}$ provided by the two edges. The velocity subspaces $V^{1}$ and $V^{2}$ are derived from the normal vectors. **(B)** Vectors ${\text{v}}_{n}^{1}$ and ${\text{v}}_{n}^{2}$ are computed by locally fitting two planes **Π**^1^ and **Π**^2^ on the events forming each edge over a space-time neighborhood. ${\text{v}}_{n}^{1}$ and ${\text{v}}_{n}^{2}$ are extracted from the slope of (respectively) **Π**^1^ and **Π**^2^ at (**p**, *t*). (extracted from Clady et al., 2015).

The geometry-based approach proposed in Clady et al. (2015) consists in collecting planes, fitted directly on the event stream (as in Benosman et al., 2014 and Section 2.1) and considered as local observations of normal visual motions, around each visual event. This event is considered as a corner event (i.e., event generates at the spatiotemporal location of a corner) if most of the collected planes intersect as a straight line in (*XYT*)^*T*^ reference frame, at a location temporally close to the event (see Figure 6).

### 3.1. Feature-based approaches

**Figure 7:**
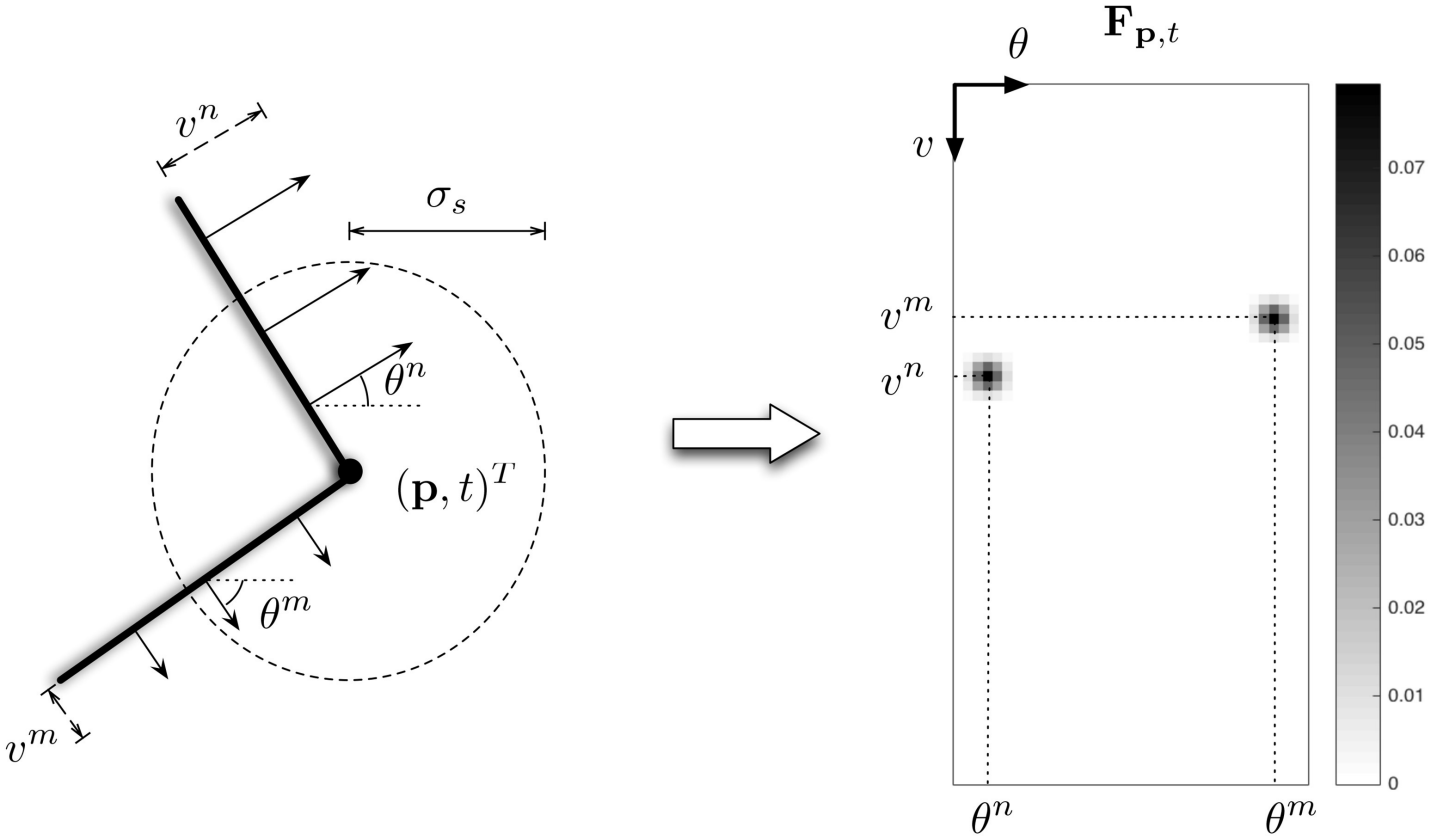
**Illustration of the feature F_**p**, ***t***_ (right figure) computed at the spatiotemporal location (p, ***t***)^***T***^ of a corner (left figure) in an ideal case**.

In the local approach, normalized **F**_**p**,*t*_ is the distribution of the normal velocities along the contours around the spatiotemporal location (**p**, *t*)^*T*^. In an ideal case illustrated in Figure 7, if this location corresponds to a corner location, **F**_**p**,*t*_ is null execpt around two velocity coordinates, (*v*^*n*^, θ^*n*^)^*T*^ and (*v*^*m*^, θ^*m*^)^*T*^, corresponding to both normal visual motions of the intersecting edges.

#### 3.1.1. 2-maxima based decision

As we can see in this Figure, detecting corners (or junctions) will consist in determining if at least two local maxima in **F**_**p**,*t*_ are present. We first propose an algorithm in order to find the two first maxima i n **F**_**p**,*t*_ consisting in:

1. finding the maximum *F*_*max*_ and its velocity coordinates ${\left(v_{max},\theta_{max}\right)}^{T}$ in **F**_**p**,*t*_,
2. inhibiting (set to zeros) all values in **F** for which the coordinates verify $|\theta^{l}-\theta_{max}|<{th}_{\theta }$, with *th*_θ_ = 20°, and
3. finding the maximum (second maximum) $F_{2^{nd}max}$ and its coordinates ${\left(v_{2^{nd}max},\theta_{2^{nd}max}\right)}^{T}$ in **F**_**p**,*t*_ previously modified in step 2.

Finally, as an isoprobabilistic representation of the intersecting edges is assumed, both values of maxima, *F*_*max*_ and $F_{2^{nd}max}$, should be close at the location of a corner (the difference would be essentially due to noise). Then we propose as selection criterion (noted $C_{2max}$) to decide if a corner is present at (**p**, *t*)^*T*^:

(10) $$\begin{array}{l} C_{2max}=\frac{F_{2^{nd}max}}{F_{max}}>{th}_{C_{2max}} \end{array}$$

with the threshold ${th}_{C_{2max}}\in \left[0,1\right]$.

#### 3.1.2. Velocity-constraint based decision

A second approach consists in considering each (*v*^*l*^, θ^*l*^)^*T*^ (or noted ${\left(v_{x}^{l},v_{y}^{l}\right)}^{T}$ in a cartesian reference frame) as a velocity constraint $V^{l}$ weighted by the value ${\text{F}}_{\text{p},t}\left(v^{l},\theta^{l}\right)$; verifying (**v**^*l*^)^*T*^**v** = ||**v**^*l*^||^2^, where $\text{v}={\left(v_{x},v_{y}\right)}^{T}$ is the velocity of the corner.

A corner is present at location (**p**, *t*)^*T*^ if **F**_**p**,*t*_ gives rise to a real solution to the equation:

(11) $$\begin{array}{l} WA\text{v}=W\text{B} \end{array}$$

where:

- $A=\left(\begin{array}{cc} v_{x}^{1} & v_{y}^{1}\\ \vdots & \vdots \\ v_{x}^{l} & v_{y}^{l}\\ \vdots & \vdots \\ v_{x}^{N_{\text{v}}} & v_{y}^{N_{\text{v}}} \end{array}\right)$, with *N*_**v**_ = *N*_*v*_*N*_θ_ the size of **F**_**p**,*t*_, i.e., the number of constraints,
- $\text{B}=\left(\begin{array}{c} ||{\text{v}}^{1}|{|}^{2}\\ \vdots \\ ||{\text{v}}^{l}|{|}^{2}\\ \vdots \\ ||{\text{v}}^{N_{\text{v}}}|{|}^{2} \end{array}\right)$ and $W=\text{diag}\left({\text{F}}_{\text{p},t}\left(v^{1},\theta^{1}\right),\ldots ,{\text{F}}_{\text{p},t}\left(v^{l},\theta^{l}\right),\ldots ,{\text{F}}_{\text{p},t}\left(v^{N_{\text{v}}},\theta^{N_{\text{v}}}\right)\right)$.

Then the over-determined system can be solved if *M* = (*WA*)^*T*^*WA* has a full rank, meaning that its two eigenvalues have to be significantly large. This significance is determined with the selection criterion established in Noble (1988):

(12) $$\begin{array}{l} C_{const}=\frac{\text{det}\left(M\right)}{\text{trace}\left(M\right)}>{th}_{C_{const}} \end{array}$$

with the threshold ${th}_{C_{const}}>0$.

Equation (11) is also solved with a least square minimization technique and solutions are considered as valid if $C_{const}$ is greater than the threshold ${th}_{C_{const}}$ usually set experimentally. Finally, a stream $S^{c}$ of corner events (including features), noted **c** = (**p**, **v**, *t*, **F**)^*T*^, is outputted.

Remark 4. *In order to be robust to noise, weak values in* **F**_**p**,*t*_ *are inhibited (associated equations are filtered out of the system): if* ${\text{F}}_{\text{p},t}\left(v^{l},\theta^{l}\right)<{th}_{\text{F}}F_{max}$ (with *th*_**F**_ ∈ [0, 1]), then $F_{\text{p},t}\left(v^{l},\theta^{l}\right)=0$.

Remark 5. *With the 2-maxima based decision approach, a corner event stream can also be obtained; the velocities of the detected corners can be estimated in a similar manner using only both maxima's coordinates, without weighting them. Furthermore, while the second approach is based on a (unnatural) mathematical analysis, the first decision method is closer to a time-based neural implementation; it could be implemented as a coincidence detector between two (or more) events, denoting the two-first (or more) maxima, outputted by the leaky integrate-and-fire neural layer assimilated to the feature* **F** *(see Discussion at the end of Section 2.3)*.

*Note that neural networks have also been proposed in the literature (Cichocki and Unbehauen, 1992) in order to solve similar systems of linear equations that are required in the velocity-constraint decision based method; VLSI implementations have even been proposed*.

Remark 6. *Note that the computation principle is quite similar to the one proposed in Clady et al. (2015); most mechanisms involved (kernels, filters, selection criteria) have been designed and set in a similar manner, in order to allow comparison in the fairest way possible (see next Section). The methods differ from each other essentially by the selection process of the velocity constraints. Through a time-based weighting process, Clady et al. (2015) considers only constraints along edges intersecting the evaluated event. The methods proposed in this article consider all the edges in a spatial neighborhood even if they are not perfectly intersecting themselves at the evaluated location; however the spatial Gaussian-based weights *w*_*s*_(·) implicitly perform a heuristic selection of the spatially closer edges, i.e., the most probable intersecting edges. So even if the location of their detected corner events should be consequently less precise, they should be close to a real corner; this is verified in the results presented in the next Section*.

### 3.2. Evaluations

**Figure 8:**
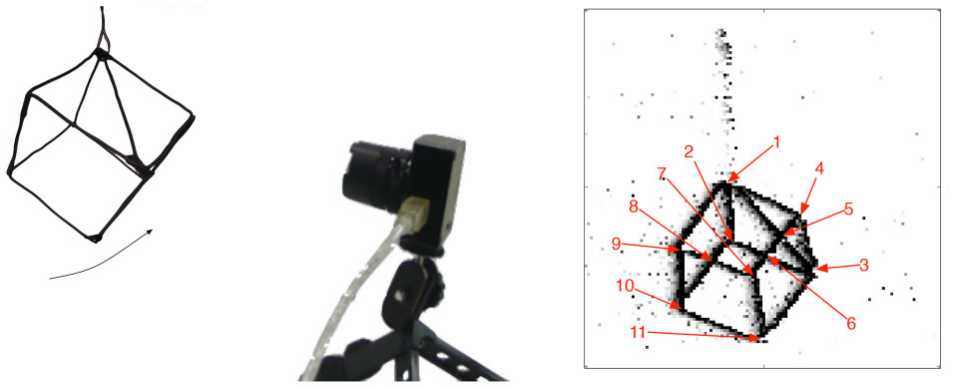
**Illustration of the experiment: a swinging 3D cube is shown to a neuromorphic camera**.

In order to evaluate the detectors, we reproduced one of the experiments proposed in Clady et al. (2015), the one with the most quantitative evaluations. It consists into a swinging wired 3D cube shown to a neuromorphic camera (DVS, see Figure 8).

**Figure 9:**
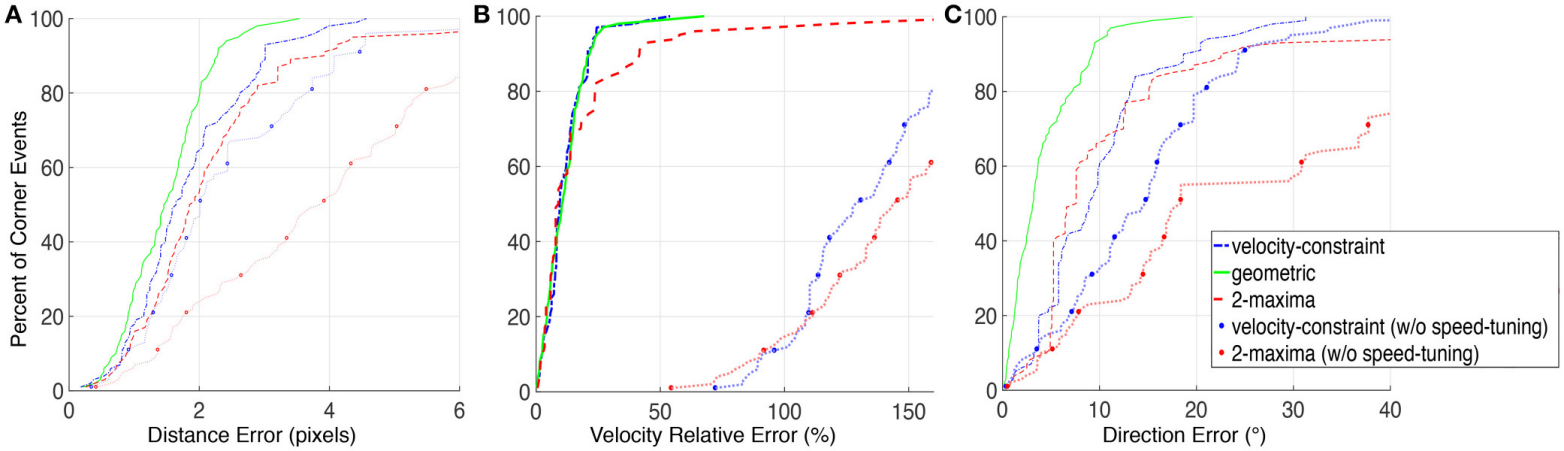
**Precision evaluation of the corner detectors; the green plain curves correspond to the results obtained with the algorithm proposed in Clady et al. (2015); the blue dash-dotted blue curves to the velocity-constraint based decision proposed in Section 3.1.2 and the red dashed curves to the 2-maxima based decision proposed in the Section 3.1.1**. The blue and red dotted curves correspond to the respective feature-based approaches but without speed-tuned temporal kernels. The left figure **(A)** represents the spatial location errors of the corner events compared to the manually-obtained ground-truth trajectories of the corners; the middle one **(B)** the relative error about the intensity of the estimated speed and the right one **(C)** its error in direction. Accuracies (X-axis fo the Figures) are given related to the considered percent (Y-axis) of the population of corner events detected with the different methods; e.g., with the method in Clady et al. (2015), 80% of the corner events have a distance error in corner location <2 pixels compared to the ground truth, see plain green curve in **(A)**.

A complete accuracy evaluation, comparing the results obtained with the geometric-based method given in Clady et al. (2015) and the methods proposed in this article, is provided in Figure 9. The corner events parameters (spatial location and velocity) and the 11 corners ones (obtained with the ground-truth) are compared using different measures of errors. Each corner event is associated to the spatially closest ground-truth corner's trajectory.

**Figure 10:**
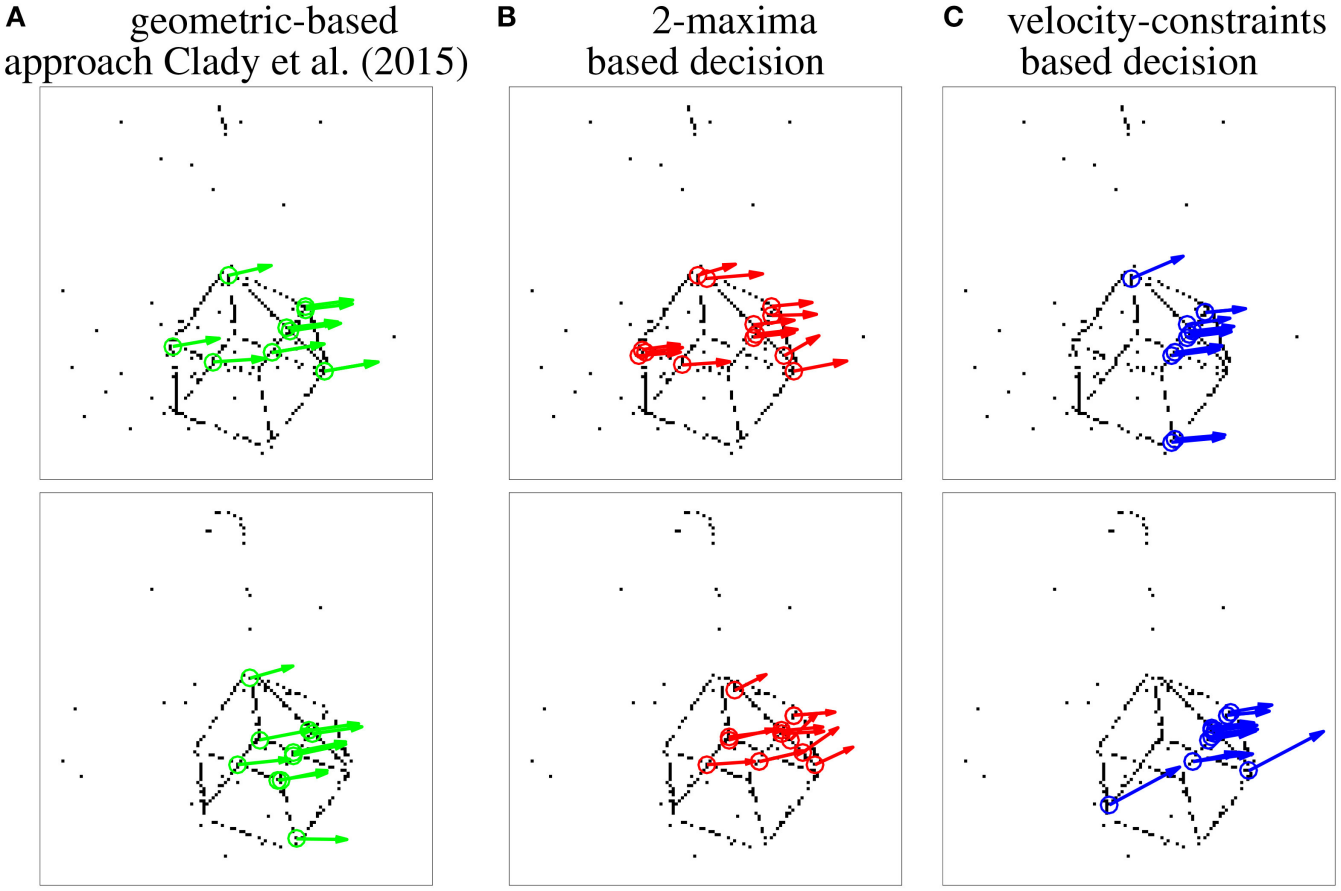
**Snapshots of the results obtained for the three compared detectors, projecting in a frame the visual events (black dots) and corner events (circles, associated to vectors representing the estimated speeds) over two short time periods (1 ms)**.

**Figure 11:**
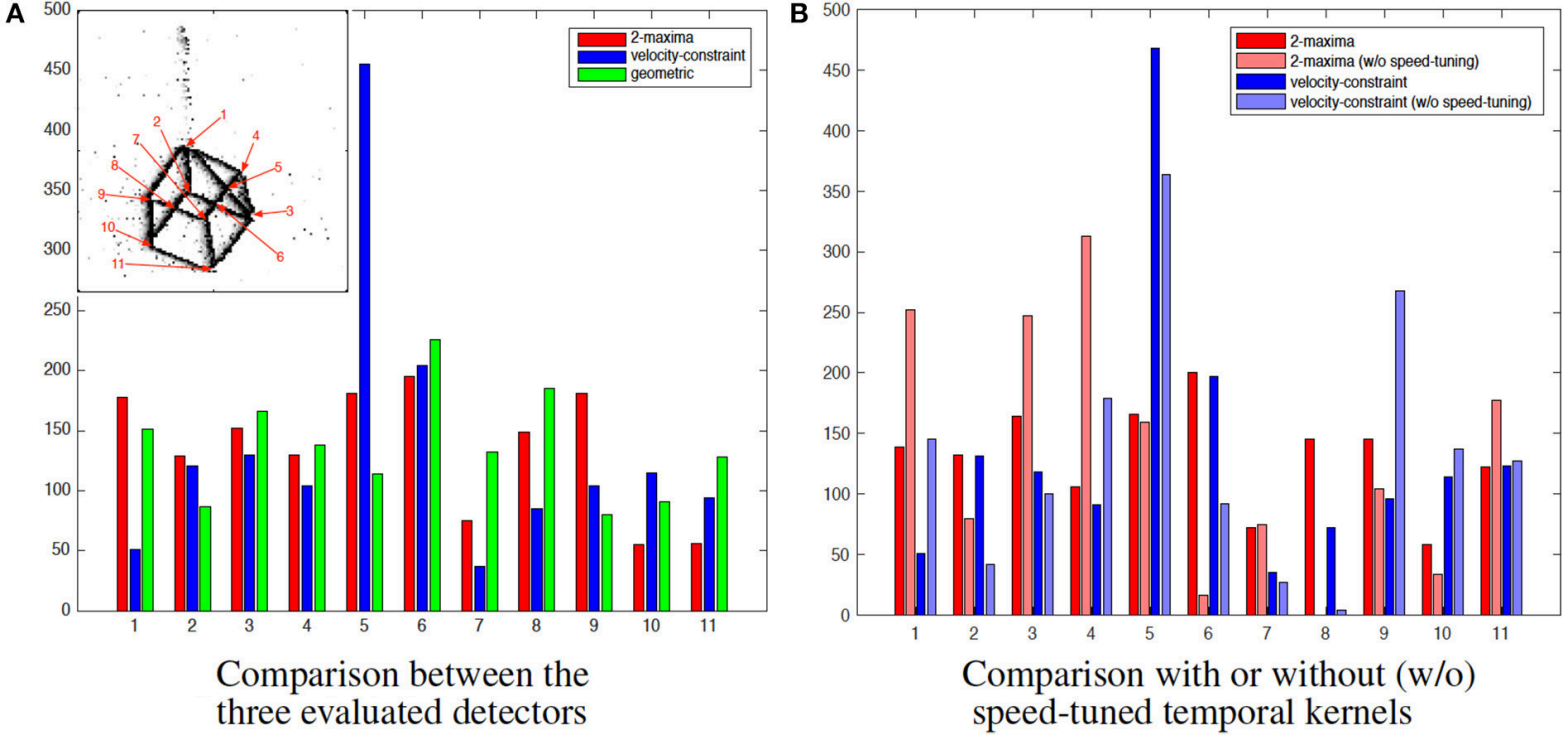
**Distributions of the detected event corners related to the labeled corners. (A)** Comparison between the three evaluated detectors. **(B)** Comparison with or without (black) speed-tuned temporal kernels.

In order to propose a fair evaluation, the thresholds used in the different methods have been set in order to detect the same number of corner events (1500) and other algorithms' parameters have been set as the ones proposed in Clady et al. (2015) (see Remark 3.6). The distribution of the corner events per corner's trajectory is shown in Figure 11A. We can observe that the distributions using the geometric-based and the 2-maxima decision based methods are closely similar. However, the one obtained with the velocity-constraint decision based method is unbalanced, with a great number (close to the third of the corner events) of detections around a particular corner, corner number 5. This can be explained by the fact that the proposed method is less spatially precise than the geometric-based one (cf. the curves in Figure 9A and Remark 3.6) and, as we can see in Figure 10, the edges around this corner generated more events than the others because they are generated by “clean” intersecting edges, see Figure 8, and then verifying well the ideal conditions for the optical flow estimation, and because it is a X-junction. It is not the case for the corners number 1, 7, and 11, for example; the high speed of the cube (close to 500*pix*.*s*^−1^, i.e., inversely close to the precision of the event timings) and their badly shaped structures (they correspond to connections between the different wires constituting the cube) make their detection very hard due to the local bad quality of the event streams (in particular, there are numerous missing events as we can see in Figure 11).

Remark 7. *Note that accuracy results in Figure 9 concern median evaluations over the 11 ground-truth corners. Each corner is associated to the spatially closest ground-truth corners trajectory. Each set of corner events (associated to a ground-truth corner) is sorted according to one of the evaluation criteria (type of errors). The Y%-most accurate corner events are then selected. Finally, the accuracy median value for this evaluation criterion is computed over all ground-truths corners. So these evaluations are *a priori* not (or weakly) biased by these differences in distributions*.

We can observe that the detectors proposed in this article are influenced by the quantification of the grid; especially in the Figure 9C representing the angular precision of the estimated speed direction. Indeed a lot of corner events have a direction-related precision close to 5°, the half of the direction-related interval length. The velocity-constrainst based decision method is less clearly influenced because it takes into account more elements in the feature (not only the elements with the maximal values, but also their neighboring elements) to estimate the speed.

In addition, Figure 11B shows the detections distribution for both feature-based methods, with or without speed-tuned temporal kernels. In the approaches without speed-tuning, the temporal decreasing factor τ has been fixed as $\tau =\frac{1}{v_{mean}}$, where *v*_*mean*_ is the mean velocity computed over all corners and the stream duration (150 ms). Without speed-tuning, some corners are not or not often detected, in particular corners number 6 and 8. They correspond to X-junctions between two intersecting edges with quite different dynamics, because generated by front and back wires. Furthermore, the accuracy performances for the approaches without speed-tuned temporal kernels are significantly lower than the ones with speed-tuned kernels, as shown in Figure 9.

Finally, if we consider that a corner event detection is valid if the distance error is <3*pixels*, the geometric-based method generates only 2% of false alarms (with a median velocity error around 10% and a median direction error around 3° for the positive detections), while this rate rises to 8% and to 18% for the velocity-constraint decision and 2-maxima decision based methods, respectively (with a median velocity error around 10% and a median direction error around 8°, for both).

We have demonstrated that the proposed feature can be used (in its local approach) to detect corners in event streams. Even if the detectors are slightly less precise and more sensitive to the quality of the event streams than the other method proposed in the literature, our feature-based approaches are more efficient in terms of memory and computation loads.

Indeed the method in Clady et al. (2015) requires to memorize the stream of the visual motion events (see Equation 2) and spatiotemporal extrapolations of them (called “normal events”) and operates quite complex computations between them. In the approach presented in this article, the visual motion events are integrated directly in the neighboring features, and corner detection related computations are operated only using the feature at the spatiotemporal location of the current event. We have measured important differences in terms of computation time between their different implementations; e.g., for the event stream used for the above evaluations, the feature-based approaches are ~10 times faster. Table 1 presents the distribution of mean computation times obtained with the different approaches and over 10 repetitions (for 1500 detections). But as the method in Clady et al. (2015) has been only implemented on Matlab (Matlab2015b), they should be taken with caution; it is indeed known that memory can be poorly managed on Matlab. Measuring the computation time without code lines dedicated to memory management (which is a crucial part of the method in Clady et al., 2015), the gain is still around 40%. While the geometric-based method is only envisioned in Clady et al. (2015) for a real time implementation on massively parallel computers such as the SpiNNaker board (see Furber et al., 2013; Orchard et al., 2015a), the feature-based approaches run in real-time on a standard computer (in C++ on a Intel Core i7-4790K @ 4GHz, using only one core and without any optimization such as integer arithmetic instead of floating point based computations, e.g., Schraudolph, 1999; Cawley, 2000) for weakly complex visual scenes such as the one presented in this study.

**Table 1:** **Distribution of mean computation times (CT) with the different approaches (estimated on Matlab2015b)**.

**Methods**	**Total CT**	***%* of CT OF estimation**	***%* of CT feature computation**	***%* of CT corner detection**
Velocity-constraint	76s.	16	83	1
2-maxima	75s.	16	83	1
Geometric	828s.	1	–	99
Geometric	132s.	9	–	91
(w/o memory management)				

Beyond this operational asset, the greatest strength of the proposed feature-based approaches lies in fact that they lead to a solution of the corner detection issue on event streams based on classical event-based neural network models (leaky integrate-and-fire neural network, coincidence detectors, etc.) as it is highlighted in Section 2.3 and Remark 5.

## 4. Application to gesture recognition

Human movement analysis is an area of study that has been quickly expanding since the 1990's (see Moeslund et al., 2006; Poppe, 2007, 2010). The evolution and miniaturization of both computers and motion capturing sensors have made motion analysis possible in a growing set of environments. They have enabled numerous applications in robotics, control, surveillance, medical purposes (Zhou and Hu, 2008) or even in video-games with the Microsoft's Kinect (Han et al., 2013). However, the available technologies and methods still present numerous limitations, discouraging their use in embedded systems. Conventional time-sampled acquisition is very problematic when implemented in mobile devices because the embedded cameras usually operate at a frame-rate of 30 to 60 Hz: normal speed gesture movements can not be properly captured. Increasing the frame rate would result in the overload of the recognition algorithm, only displacing the bottleneck from acquisition to post-processing. Furthermore, conventional cameras and infrared-based methods are perturbed by dynamic lighting and infra-red radiations emitted by the sun (cf. Panaïté et al., 2011). Because they both require light-controlled environments, those technologies are unsuitable for outdoor use.

Asynchronous event-based sensing technology is expected to overcome several limitations encountered by state-of-the-art gesture recognition systems, in particular for battery-powered, mobile devices. These vision sensors, due to their near continuous-time operation, allow capturing the complete and true dynamics of human motion during the whole gesture duration. Due to the pixel-individual style of acquisition and pre-processing of the visual information, and in contrast to practically all existing technologies, they will be also able to support device operation under uncontrolled lighting conditions, particularly in outdoor scenarios (cf. Simon-Chane et al., 2016). Native redundancy suppression performed in event-based sensing and processing will ensure that computation can be performed in real time, while at the same time saving energy, decreasing system complexity.

Gesture recognition using neuromorphic camera has already been investigated by Lee et al. (2014). A stereo pair of DVS allows them to compute disparity in order to cluster the hand. Then, they use a tracking algorithm to extract the 2D trajectory of the movement. Finally the trajectory is sampled into directions, and the obtained sequence of directions is fed to a HMM classifier. This approach uses event-based information only during the first step (extraction of the location of the hand). In addition, with this type of multi-steps architecture, a failure in a step could result in the failure of the whole system.

Here we propose to demonstrate that our feature can be used to detect and recognize more directly gestures. Hoof-like features (see Section 4.1) are derived from the feature matrix and provided to a classification architecture that performs simultaneously detection and recognition. It is based on hybrid generative/discriminative classifiers (Lasserre et al., 2006) in order to associate at each feature its probabilities to belong to the considered (hand) gestures or not, and these probabilities are integrated over time through a network of Bayes filters (Thrun et al., 2008).

### 4.1. A more compact and invariant representation

In order to reduce the dimensionality of the feature (it is often required in machine learning, in order to address the “curse of dimensionality” issue) and to provide (global speed- and) scale-invariance property to the gesture representation, **F** can be transformed into a more compact representation, noted **h** (**h**_**p**,*t*_ or **h**_*t*_, in local or global approaches, respectively) and named hoof-like in reference to the Histogram of Oriented Optical Flow (HOOF) introduced by Chaudhry et al. (2009) in frame-based vision. This transformation consists in summing the intensities of the optical flow vectors with respect to their directions.

From the feature **F**, ${\text{h}}_{\text{p},t}={\left[h_{\text{p},t;1},\ldots .,h_{\text{p},t;N_{\theta }}\right]}^{T}$ can be easily obtained:

(13) $$\begin{array}{l} h_{\text{p},t;i}=\underset{k}{\sum }v^{k}{\text{F}}_{\text{p},t}\left(v^{k},\theta^{i}\right) \end{array}$$

In the global approach, normalization (to sum to 1) makes the hoof-like feature globally speed- and scale-invariant. Figure 4D represents the histogram of oriented optical flows computed globally on an event stream capturing a walking human (Figure 4A).

### 4.2. Classification architecture

We propose a classification architecture where the problem is framed as a Bayes filter, that is estimating the probabilities of gestures recursively over time using incoming measurements, given as the hoof-like features ${\text{h}}_{t_{0}:t_{k}}\in H$ computed globally from every visual events [**e**_0_, **e**_*k*_].

Then we note the state $g^{i}\in G$, the gesture (numerated *i*, *i* ∈ [1, *K*]) that the user is currently performing. A state *g*^0^ is added in G, in order to consider the not-considered gestures or the instants while the user is not performing a hand gesture.

The camera observes the user's action and at each occurring feature estimates a distribution over the current state $g_{t_{k}}^{i}$:

(14) $$\begin{array}{l} p\left(g_{t_{k}}^{i}|{\text{h}}_{t_{0}:t_{k}}\right) \end{array}$$

where ${\text{h}}_{t_{k}}\in H$ is the observation of the gesture occurring at time *t*_*k*_.

To estimate this probability, a time update and a measurement update are performed alternately. The time update updates the belief that the user is performing a specific gesture given previous information:

(15) $$\begin{array}{l} p\left(g_{t_{k}}^{i}|{\text{h}}_{t_{0}:t_{k-1}}\right)=\underset{g_{k-1}^{j}\in G}{\sum }p\left(g_{t_{k}}^{i}|g_{t_{k-1}}^{j}\right)p\left(g_{t_{k-1}}^{i}|{\text{h}}_{t_{0}:t_{k-1}}\right) \end{array}$$

The time update includes a transition probability from the previous state to the current state. As no-contextual information is available here, we assume that an user is likely to perform the same gesture, and at each timestamp has a large probability of transitioning to the same state:

(16) $$p(g_{t_{k}}^{i}|g_{t_{k-1}}^{j})=\{\begin{array}{ll} \frac{1}{|G|}+\frac{|G|-1}{|G|}\exp\left(-\frac{t_{k}-t_{k-1}}{\tau_{g}}\right) & \text{if }i=j\\ \frac{1}{|G|}-\frac{1}{|G|}\exp\left(-\frac{t_{k}-t_{k-1}}{\tau_{g}}\right) & \text{otherwise} \end{array}$$

with τ_*g*_ set to 150 ms, less than the half duration of shorter gestures. This assumption means that the gesture's certainty slowly decays over time, in the absence of corroborating information, converging to a uniform distribution (even if no event is observed).

The measurements update combines the previous belief with the newest observation to update each belief state, such as:

(17) $$\begin{array}{l} \begin{array}{cc} p\left(g_{t_{k}}^{i}|{\text{h}}_{t_{0}:t_{k}}\right) & =\frac{p\left({\text{h}}_{t_{k}}|g_{t_{k}}^{i}\right)p\left(g_{t_{k}}^{i}|{\text{h}}_{t_{0}:t_{k-1}}\right)}{p\left({\text{h}}_{t_{k}}|{\text{h}}_{t_{0}:t_{k}}\right)}\\ \propto p\left({\text{h}}_{t_{k}}|g_{t_{k}}^{i}\right)p\left(g_{t_{k}}^{i}|{\text{h}}_{t_{0}:t_{k-1}}\right) \end{array} \end{array}$$

In order to estimate $p\left({\text{h}}_{t_{k}}|g_{t_{k}}^{i}\right)$, we propose a machine learning based approach to compute and select generative models for gesture. It is decomposed into two steps:

- For the first step, we collect hoof-like features computed while the users (included in the training database, see Section 4.3.1) performed a gesture *g*^*i*^, *i* ∈ [1, *K*]. Then a k-means algorithm is applied on them in order to compute *N* candidate models, noted **m***^g^i^^*.
- The second step consists in selecting from these candidate models, the ones that are the most discriminative against hoof-like features collected from the rest of the training event streams; these last features have been computed during other considered gestures (*g*^*j*^ with *i* ≠ *j*) or during other period times when users were not performing gestures. This selection is processed through a discrete Adaboost classifier.

Adaboost (Freund and Schapire, 1996) is an iterative algorithm that finds, from a feature set, some weak but discriminative classification functions and combines them in a strong classification function:

(18) $$B=\{\begin{array}{l} \begin{array}{l} 1\text{, }\sum_{s=1}^{S}\lambda_{s}b_{s}\geq \frac{1}{2}\sum_{s=1}^{S}\lambda_{s},\\ -1\text{, otherwise}, \end{array} \end{array}$$

where *B* and *b* are the strong and weak classification functions, respectively, and λ is a weight coefficient for each *b*. *T* is the threshold of the strong classifier *B*. The principle of the Adaboost algorithm is to select, at each iteration, a new weak classifier in favor of the instances (or features) misclassified by previous classifiers, through a weighting process attributing more influence to misclassified instances.

Note that a threshold value, noted *th*_*B*_, can be defined (such as the condition in Equation 18 can be written: $\frac{2}{\overset{S}{\underset{s=1}{\sum }}\lambda_{s}}\overset{S}{\underset{s=1}{\sum }}\lambda_{s}b_{s}\geq th_{B}$) in order to optimize a particular classification performance. During the learning step, its default value is 1; this means a classification frontier at the middle of the margin (see Schapire et al., 1998). Increasing or reducing its value correspond to moving the frontier closer or further to the positive class, respectively.

In literature, discriminative training of generative models, as we propose here, has been shown as efficient learning methods in numerous applications as object or human detection (Holub et al., 2005; Negri et al., 2008; Wang et al., 2011), face or character recognition (Prevost et al., 2005; Grabner et al., 2007) or for medical purposes (Deselaers et al., 2008; Wang et al., 2015). The proposed classifier based on the training and the selection of generative models in a discriminative way, combines indeed the main characteristics of discriminative and generative approaches: discriminative power and generalization ability, respectively. The latter is in particular very important in our application, when a weak amount of labeled training data is available, see Section 4.3.1.

Following the framework described in Jing et al. (2008), we propose to design weak classifiers as generative ones, associated to each candidate models ${\text{m}}_{s}^{g^{i}}$ (*s* ∈ [1, *N*]):

(19) $$b_{s}^{i}=\{\begin{array}{l} \begin{array}{l} 1,\text{ if }f(h,m_{s}^{g^{i}})=\exp\left(-\frac{d{\left(h,m_{s}^{g^{i}}\right)}^{2}}{\theta_{s}^{g^{i}}}\right)\geq \frac{1}{2}\\ -1,\text{ otherwise}, \end{array} \end{array}$$

where *d*(·, ·) is the Euclidean distance and $\theta_{s}^{g^{i}}$ parametrizes the likelihood function *f* and is computed at each iteration of the algorithm through a maximum-likelihood estimation (taking into account the weights attributed to features).

During training, Adaboost based algorithm tends to select iteratively the most discriminative and complementary models for each gesture. We limit the number of selected models, such as the relative difference between F-measure (computed on training database, see Section 4.3.1) obtained at the corresponding iteration is superior or equal to 95% of its maximum (obtained with a greater number of iterations of Adaboost algorithm). Let us remind that F-measure is defined as $2\times \frac{\text{precision}\times \text{recall}}{\text{precision}+\text{recall}}$. Optimizing it means also to determine a number of models for which an acceptable compromise between precision (the ratio of positive detections to instances belonging to performed gestures) and recall (the ratio of positive detections to all instances detected as belonging to gestures) is reached.

The probability $p\left({\text{h}}_{t_{k}}|g_{t_{k}}^{i}\right)$ is then estimated as proportional to a measure (∈ [0, 1]) operated between the hoof-like feature and the set of selected models (applying a sigmoidal function to the output of the strong classifier):

(20) $$\begin{array}{l} p\left({\text{h}}_{t_{k}}|g_{t_{k}}^{i}\right)\propto L\left({\text{h}}_{t_{k}},g^{i}\right)=\frac{1}{1+\exp\left(\frac{2}{\overset{S^{i}}{\underset{s=1}{\sum }}\lambda_{s}^{i}}\overset{S^{i}}{\underset{s=1}{\sum }}\lambda_{s}^{i}b_{s}^{i}-{th}_{B}^{i}\right)} \end{array}$$

with *i* ∈ [1, *K*] and ${th}_{B}^{i}$ is the threshold obtained optimizing the F-measure. The probability associated to not-considered gesture (or no-gesture), noted *g*^0^, is then defined as:

(21) $$\begin{array}{l} p\left({\text{h}}_{t_{k}}|g_{t_{k}}^{0}\right)\propto 1-{\text{max}}_{i\in \left[1,K\right]}\left(L\left({\text{h}}_{t_{k}},g^{i}\right)\right) \end{array}$$

**Figure 12:**
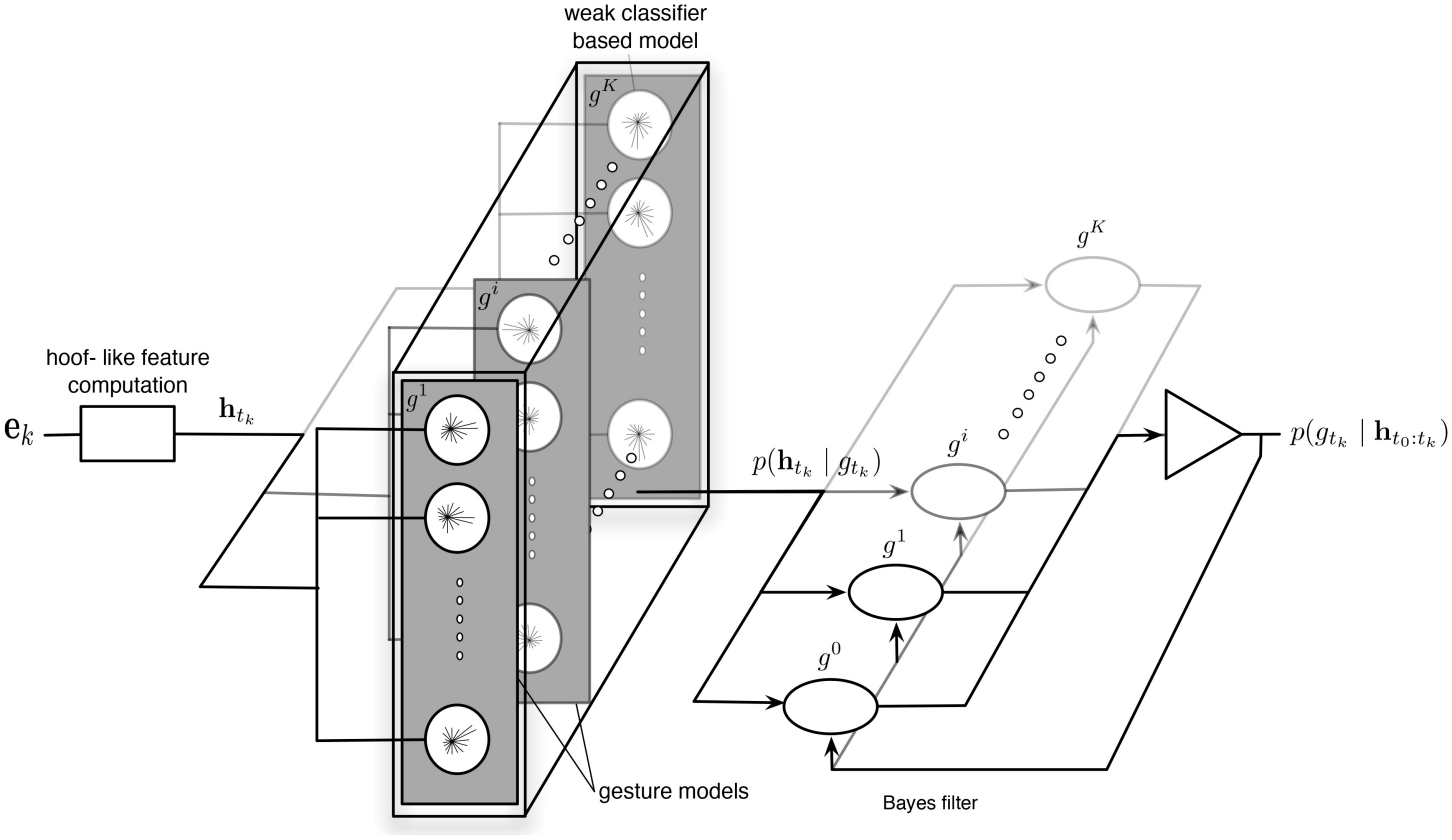
**Gesture Recognition Architecture: for each occurring hoof-like feature h_*t*_*k*__, the distribution of probabilities noted *p*(h_*t*_*k*__ ∣ *g*_*t*_*k*__), is estimated comparing the features to models computed and selected through an Adaboost-based learning process**. Then the probabilities of gestures, noted *p*(*g*_*t*_*k*__ ∣ **h**_*t*_0_:*t*_*k* − 1__), are estimated recursively over time.

Figure 12 presents the obtained classification architecture. Finally a gesture's class *G*_*t*_*k*__ at each time is attributed from the distribution of probabilities, defined as:

(22) $$\begin{array}{l} G_{t_{k}}={\text{argmax}}_{i\in \left[0,K\right]}\left(p\left(g_{t_{k}}^{i}|{\text{h}}_{t_{0}:t_{k}}\right)\right) \end{array}$$

Remark 8. *Even if our implementation is based on a learning process not directly related to neural approaches (essentially due to the limited size of the database), we can observe that the resulting classification architecture could be fully implemented in an event-based framework. Through a rate-coding model, hoof-like features could be computed and transmitted from the leaky integrate-and-fire neural network, corresponding to the feature computation, as evoked in Section 2.3, to neural networks performing their comparison with gesture models (considering maybe another distance than the Euclidean one used here) and outputting positive events when they match; these positive events corresponding to the weak classifier responses ($b_{s}^{i}$). The coefficients $\lambda_{s}^{i}$ would be then assimilated to synaptic weights. The other operations, in particular involved in Bayes filters, would correspond to feedback lines and basic mathematical operations that can be modeled using precise timing and event-based paradigms as demonstrated in Lagorce and Benosman (2015)*.

### 4.3. Results

#### 4.3.1. Experimental protocol

The protocol assumes that the users performed gestures in front of the camera. Event streams (using the ATIS camera) have been collected with nine users (young and middle-aged people working in the laboratory). All users are right-handed but the database could be extended to left-handed users by mirroring the sequences horizontally.

**Figure 13:**
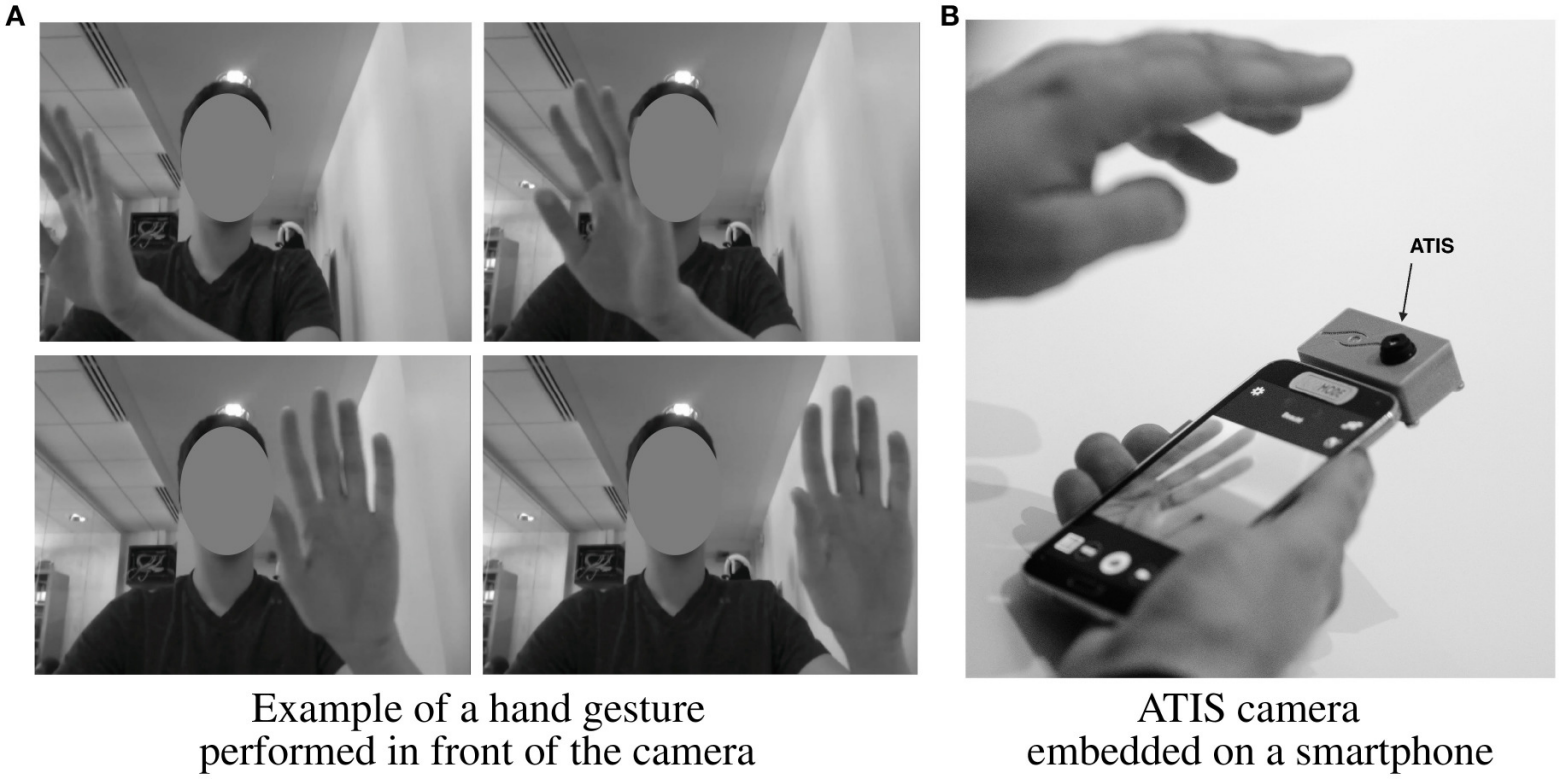
**Illustration of the targeted human-machine interaction. (A)** Example of a hand gesture performed in front of the camera. **(B)** ATIS camera embedded on a smartphone.

The hand is moving at a distance around 30 cm from the camera, approximatively. Note that this distance has been determined to ensure that the hand is fully viewed by the camera (see Figure 13A) considering the current optic lens (this distance should be reduced when a wider-angle lens will be implemented). Each gesture is repeated five times by each user, varying the hand speed.

**Figure 14:**
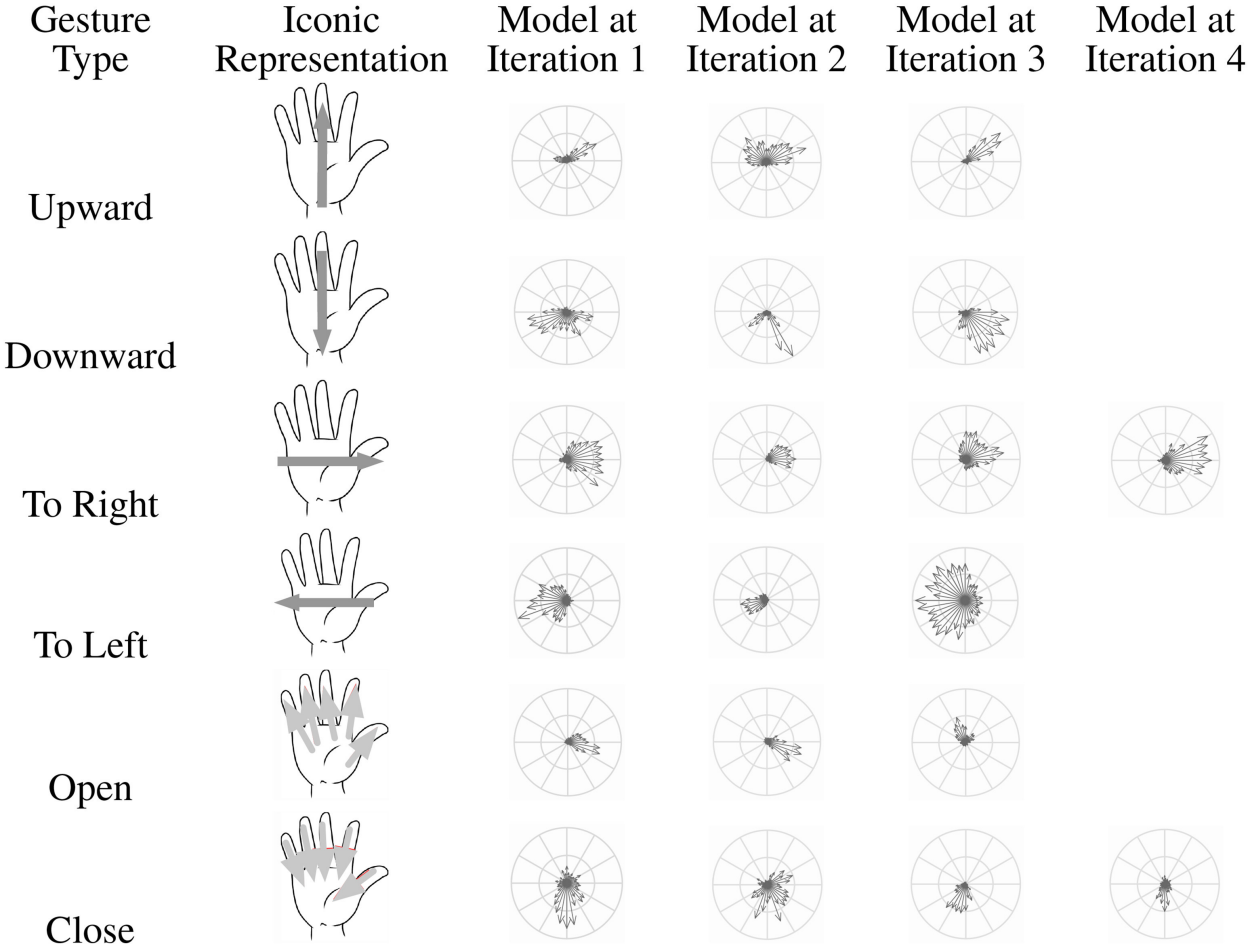
**Iconic representations (second column) of the gestures (first column) and corresponding models selected by the Adaboost-based machine learning process**.

Six gestures have been defined and correspond to a dictionary of coarse gestures; the gesture is defined by the global motion of the hand (hand moving to the left, to the right, upward, downward, opening, or closing). These gestures could match with the main controls we could intend to execute interacting with a smartphone or a tablet (navigating in a menu or a list, selecting/unselecting an object or an application), i.e., the targeted application (see Figure 13B). Furthermore, they constitute a dictionary for more complex gestures, successively combining these movements. In Figure 14, an iconic representation of these coarse gestures is presented in the second column.

The training database is composed of the event streams collected with five users and the test database with the four other ones. During the evaluations (see next Section), a cross-validation is performed ten times (presented evaluations are the obtained mean values), putting randomly the users in the training or test databases. 30, 000 hoof-like features, computed on the training streams, are collected randomly and equitably in the time periods when gestures are performed (including the not-considered gestures or no gesture class) to train the Adaboost classifiers with a *one*-vs.*-all* strategy. An equal quantity is again randomly selected for the F-measure based optimization process and the selection of the number of models. Six hundred candidate models per gesture have been computed using k-means algorithm. The characteristics of the hoof-like features are the same as described in Section 2.2 (*N*_θ_ = 36, etc).

A gesture is considered as detected when the duration of a time period with classified gestures (*G*_*t*_*k*__ ≠ 0 in Equation 22) is over 300 ms. This detection is counted as positive if this time period overlaps the manually labeled ground truth (with an overlap ratio superior to 0.5).

#### 4.3.2. Evaluations

Figure 14 represents the considered gestures and the models selected by Adaboost during a learning process (see Section 4.2). We can observe that the number of selected models is relatively weak (3 or 4). This means that the hoof-like features are able to represent well the gestures despite their (speed- and user-related) variability, mostly thanks to its speed- and scale-invariance property.

Another observation concerns the “shape” of the feature models. For most of them, they match well to the iconic representation of the corresponding motion; for example, for the motions to the left and to the right, most speed vectors are oriented to these respective directions, etc. However, some singularities have to be explained considering not only the global motion but also the directions of the principal contours of the human parts (hand, finger and arm) involved in the hand movement. For the opening hand motion, models obtained at iterations 1 and 2 highlight the motion of the thumb, for which the moving contours are prevalent in the feature. For the downward motion, the contours of the arm are too prevalent (see models obtained at iterations 2 and 3) because the camera viewed the user's bust (see Figure 13).

In terms of detection performance, we obtained a mean precision of 91% and a mean recall of 83% (F-measure = 0.85) which confirm the great discrimination power of the proposed feature. Note that the F-measures obtained during the optimization (to determine *th*_*B*_ and the number of models) are around 0.75. The greater value obtained at the final output highlights the filtering action of the Bayes filters.

**Figure 15:**
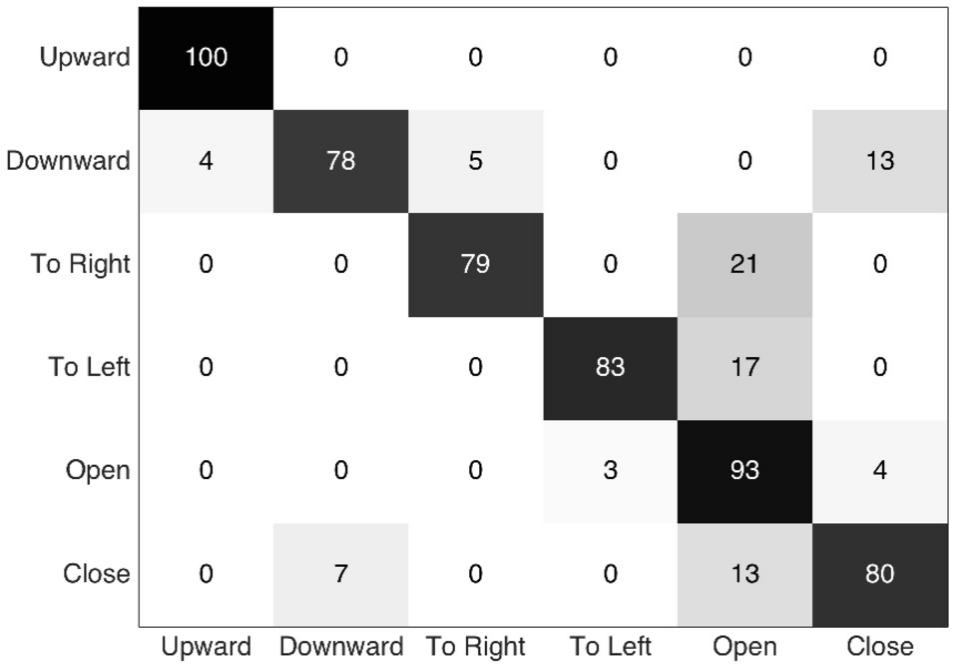
**Confusion matrix (expressed in percent) showing the recognized gestures (columns) related to the performed gestures (lines), among the positive detections**.

Finally the confusion matrix given in Figure 15 shows us the recognized gestures among the positive detections. The downward and closing hand gestures are obviously a little confused because the similarity of the hand's and the fingers' motions, respectively. The confusion of other gestures with the opening hand is probably due to the fact that the gesture is hard to detect, probably because the larger proportion of the movement involved the other fingers than the thumb and their moving contours generated few visual events (because in folded positions; the finger-skin vs. palm-skin contrast changes are weakly captured, see Remark 2.2). Indeed, in order to optimize the F-measure, the proposed process tends to select a low threshold compared to others (3 or 4 times lower); this means that classification frontier defined for this gesture tends to include other gestures. Hence, these gestures are sometimes misclassified as opening hand.

In further developments, we expect to improve these performances combining this global feature with locally computed ones, taking into account their relative spatio-temporal relationships. This should help us to better distinct the global motion of the hand and the local motions of the fingers, and hence better detect and categorize gestures.

## 5. Conclusion and discussion

In this article, we have proposed a motion-based feature for event-based vision. It consists in encoding the local or global visual information provided a neuromorphic camera in a grid-sampled map of optical flow. Collecting optical flow (or visual motion events) computed around each visual event in a neighborhood or in the entire retina, this map represents their current probabilistic distribution in a speed- and direction-coordinates frame.

Two event-based pattern recognition frameworks have been developed in order to demonstrate its usefulness for such tasks. The first one is dedicated to detection of specific interest points, corners. Two feature-based approaches have been developed and evaluated. Formulated as an intersection of constraints issue, this fundamental task in computer vision can be resolved operating with the information encoded in the proposed local feature. The second one consists in a hand gesture recognition system for human-machine interaction, in particular with mobile devices. More compact and scale-invariant representations (called hoof-like features) of the motion observed in the visual scene, are extracted directly from the global version of the proposed feature, and feed a classification architecture, based on a discriminative learning schema of gestures' generative models and framed as a Bayes filter. Evaluations show that this feature has sufficient descriptive power to solve such pattern recognition problems. Other extensions or derivations of the proposed feature can be also envisioned in further developments, in order to address other pattern recognition issues. For example, summing the elements of the feature, with respect to their directions and without weighting them by corresponding speed, will result into another compact form, similar to the hog (histogram of oriented gradients) feature proposed by Dalal and Triggs (2005). This feature and its derivations have been demonstrated as very efficient for many pattern recognition tasks in frame-based vision. To evaluate it in event-based vision would required to design event-based and dedicated classification architecture(s).

It is interesting to notice that our motion-based feature allows us to detect features defined by “static” properties, i.e., corners, and recognize dynamic actions, i.e., gestures, in visual scenes. All required information for both tasks are provided by a local computation of optical flow; this information is precisely encoded in the primary area (V1) of the visual cortex via the selectivity of V1 neurons. We underline also that the proposed frameworks are fully incremental and could be implemented as event-based neural networks, in particular thanks to speed and direction coordinates frame based representation of the visual motion information.

Such polar coordinate frame based representations have been already investigated for computer vision; e.g., based on bank of Gabor filters, using whether synchronous frame-based (Lades et al., 1993; Jain et al., 1997; Lyons et al., 1998, etc.) or asynchronous event-based (Akolkar et al., 2015) visual information. Works about natural image statistics (Hyvarinen et al., 2009) showed that similar decompositions of visual information emerge naturally from independent component analysis applied on patches collected on natural images. Recently, a work in Chandrapala and Shi (2016) encoding more directly local event streams as local spatiotemporal surfaces (Lagorce et al., 2016), showed that an unsupervised learning process applied on a relatively large database acquired with a neuromorphic camera, leads to a similar result: basic and local feature extractors coding contours' speed and direction. Moreover, other works (Cedras and Shah, 1995; Chaudhry et al., 2009; Ahad et al., 2012, etc.) in frame-based vision have shown that optical flow is a valuable information to encode in features for pattern recognition tasks.

In addition, the work presented in this article supports the proposition that optical flow's speed and direction based grid is not only a powerful manner for encoding visual information in pattern recognition tasks, but it plays also a key role at a computational level when dealing with asynchronous event-based streams. Indeed we have shown that, to compute the distribution of optical flow along current edges, we need to take into account their respective dynamics, in order to ensure that the moving edges are equitably represented in the feature (whatever their own dynamics). The discretization of the visual motion information into the proposed speed- and direction-based grid allows us to incorporate directly the required speed-tuned temporal kernels in the structure of the computational architecture computing the feature. We have in addition proposed that this architecture can be implemented as a leaky integrate-and-fire neural layer, wherein neurons have then speed-tuned integration times; so it could be further integrated as the first layer in a spiking neural network using back-propagation based deep learning technique, as the one recently proposed by Lee et al. (2016) wherein LIF neurons are also used.

Finally, in the asynchronous event-based multilayer architectures proposed recently in Chandrapala and Shi (2016) and Lagorce et al. (2016), the integration times are tuned as increasing at higher layers. In addition, in our gesture recognition architecture, we have set the integration time in Bayes filters regarding the gesture durations, not the dynamics of the visual information. Further, investigations could address the following issue: when (or at what level in hierarchical models) the integration times should be tuned not regarding the dynamics of the perceived information, but other temporal considerations or dynamics, maybe related to a targeted task or action, or maybe related to other perceptive, learning, or memory functions.

## Author contributions

XC developed the theory for feature and performed experiments and analysis for corner detection. XC, JM, and SB designed the experiments, performed analysis and interpreted data for gesture recognition. XC wrote the article and JM, SB, and RB helped to edit the manuscript.

### Conflict of interest statement

The authors declare that the research was conducted in the absence of any commercial or financial relationships that could be construed as a potential conflict of interest.

## References

[B1] Adelson E. Movshon J. (1982). Phenomenal coherence of moving visual patterns. Nature 200, 523–525. 10.1038/300523a0 7144903

[B3] Ahad M. A. R. Tan J. K. Kim H. Ishikawa S. (2012). Motion history image: its variants and applications. Mach. Vis. Appl. 23, 255–281. 10.1007/s00138-010-0298-4

[B2] Akolkar H. Meyer C. Clady Z. Marre O. Bartolozzi C. Panzeri S. Benosman R. (2015). What can neuromorphic event-driven precise timing add to spike-based pattern recognition? Neural Comput. 27, 561–593. 10.1162/NECO_a_00703 25602775

[B4] Bair W. Movshon J. A. (2004). Adaptive temporal integration of motion in direction-selective neurons in macaque visual cortex. J. Neurosci. 24, 7305–7323. 10.1523/JNEUROSCI.0554-04.2004 15317857 PMC6729763

[B5] Benosman R. Clercq C. Lagorce X. Ieng S.-H. Bartolozzi C. (2014). Event-based visual flow. IEEE Trans. Neural Netw. Learn. Systems 25, 407–417. 10.1109/TNNLS.2013.2273537 24807038

[B6] Brosch T. Tschechne S. Neumann H. (2015). On event-based optical flow detection. Front. Neurosci. 9:137. 10.3389/fnins.2015.00137 25941470 PMC4403305

[B7] Camuñas-Mesa L. A. Serrano-Gotarredona T. Ieng S. H. Benosman R. B. Linares-Barranco B. (2014). On the use of orientation filters for 3d reconstruction in event-driven stereo vision. Front. Neurosci. 8:48. 10.3389/fnins.2014.00048 24744694 PMC3978326

[B8] Carneiro J. Ieng S.-H. Posch C. Benosman R. (2013). Event-based 3d reconstruction from neuromorphic retinas. Neural Netw. 45, 27–38. 10.1016/j.neunet.2013.03.006 23545156

[B9] Cawley G. C. (2000). On a fast, compact approximation of the exponential function. Neural Comput. 12, 2009–2012. 10.1162/089976600300015033 10976136

[B10] Cedras C. Shah M. (1995). Motion-based recognition: a survey. Image Vis. Comput. 13, 129–155. 10.1016/0262-8856(95)93154-K

[B11] Censi A. Strubel J. Brandli C. Delbrück T. Scaramuzza D. (2013). Low-latency localization by active led markers tracking using a dynamic vision sensor, in IEEE/RSJ International Conference on Intelligent Robots and Systems (IROS) (Tokyo: IEEE), 891–898.

[B12] Chandrapala T. N. Shi B. E. (2016). Invariant feature extraction from event based stimuli. arXiv:1604.04327.

[B13] Chaudhry R. Ravichandran A. Hager G. Vidal R. (2009). Histograms of oriented optical flow and binet-cauchy kernels on nonlinear dynamical systems for the recognition of human actions, in IEEE Conference on Computer Vision and Pattern Recognition, 2009. CVPR 2009. (Miami, FL: IEEE), 1932–1939.

[B14] Cichocki A. Unbehauen R. (1992). Neural networks for solving systems of linear equations and related problems. IEEE Trans. Circ. Syst. I Fundam. Theor. Appl. 39, 124–138.

[B15] Clady X. Clercq C. Ieng S.-H. Houseini F. Randazzo M. Natale L.. (2014). Asynchronous visual event-based time-to-contact. Front. Neurosci. 8:9. 10.3389/fnins.2014.00009 24570652 PMC3916774

[B16] Clady X. Ieng S.-H. Benosman R. (2015). Asynchronous event-based corner detection and matching. Neural Netw. 66, 91–106. 10.1016/j.neunet.2015.02.013 25828960

[B17] Dalal N. Triggs B. (2005). Histograms of oriented gradients for human detection, in IEEE Computer Society Conference on Computer Vision and Pattern Recognition (CVPR'05), Vol. 1 (San Diego, CA: IEEE), 886–893.

[B18] Debaecker T. Benosman R. Ieng S. H. (2010).“Image sensor model using geometric algebra: from calibration to motion estimation,” in Geometric Algebra Computing, eds Bayro-Corrochano E. Scheuermann G. (London: Springer-Verlag), 277–297.

[B19] Delbrück T. Lang M. (2013). Robotic goalie with 3 ms reaction time at 4% cpu load using event-based dynamic vision sensor. Front. Neurosci. 7:223. 10.3389/fnins.2013.00223 24311999 PMC3836084

[B20] Delbrück T. Linares-Barranco B. Culurciello E. Posch C. (2010). Activity-driven, event-based vision sensors, in Proceedings of 2010 IEEE International Symposium on Circuits and Systems (ISCAS) (Paris), 2426–2429.

[B21] Deselaers T. Heigold G. Ney H. (2008). Svms, gaussian mixtures, and their generative/discriminative fusion, in 19th International Conference on Pattern Recognition. ICPR 2008 (Tampa, FL: IEEE), 1–4.

[B22] Dickscheid T. Schindler F. Förstner W. (2011). Coding images with local features. Int. J. Comput. Vis. 94, 154–174. 10.1007/s11263-010-0340-z

[B23] Drazen D. Lichtsteiner P. Häfliger P. Delbrück T. Jensen A. (2011). Toward real-time particle tracking using an event-based dynamic vision sensor. Exp. Fluids 51, 1465–1469. 10.1007/s00348-011-1207-y

[B24] Firouzi M. Conradt J. (2015). Asynchronous event-based cooperative stereo matching using neuromorphic silicon retinas. Neural Process. Lett. 43, 311–326. 10.1007/s11063-015-9434-5

[B25] Freund Y. Schapire R. E. (1996). Experiments with a new boosting algorithm, in Icml, ed Kaufmann M. (Bari), Vol. 96, 148–156.

[B26] Furber S. Lester D. Plana L. Garside J. Painkras E. Temple S. (2013). Overview of the spinnaker system architecture. IEEE Trans. Comput. 62, 2454–2467. 10.1109/TC.2012.142

[B27] Gauglitz S. Höllerer T. Turk M. (2011). Evaluation of interest point detectors and feature descriptors for visual tracking. Int. J. Comput. Vis. 94, 335–360. 10.1007/s11263-011-0431-5

[B28] Gerstner W. Kistler W. M. (2002). Spiking Neuron Models: Single Neurons, Populations, Plasticity. Cambridge University Press.

[B29] Gil A. Mozos O. M. Ballesta M. Reinoso O. (2010). A comparative evaluation of interest point detectors and local descriptors for visual slam. Mach. Vis. Appl. 21, 905–920. 10.1007/s00138-009-0195-x

[B30] Grabner H. Roth P. M. Bischof H. (2007). Eigenboosting: combining discriminative and generative information, in 2007 IEEE Conference on Computer Vision and Pattern Recognition (Minneapolis, MN: IEEE), 1–8.

[B31] Han J. Shao L. Xu D. Shotton J. (2013). Enhanced computer vision with microsoft kinect sensor: a review. IEEE Trans. Cybernet. 43, 1318–1334. 10.1109/TCYB.2013.2265378 23807480

[B32] Harris C. Stephens M. (1988). A combined corner and edge detector, in Proceedings of the 4th Alvey Vision Conference (Manchester), 147–151.

[B33] Holub A. D. Welling M. Perona P. (2005). Combining generative models and fisher kernels for object recognition, in Tenth IEEE International Conference on Computer Vision (ICCV'05), Vol. 1 (Beijing: IEEE), 136–143.

[B34] Hubel D. H. Wiesel T. N. (1962). Receptive fields, binocular interaction and functional architecture in the cat's visual cortex. J. Physiol. 160, 106–154. 14449617 10.1113/jphysiol.1962.sp006837 PMC1359523

[B35] Hubel D. H. Wiesel T. N. (1968). Receptive fields and functional architecture of monkey striate cortex. J. Physiol. 195, 215–243. 4966457 10.1113/jphysiol.1968.sp008455 PMC1557912

[B36] Hyvarinen A. Hurri J. Hoyer P. O. (2009). Natural Image Statistics: A Probabilistic Approach to Early Computational Vision. Springer.

[B37] Jain A. K. Ratha N. K. Lakshmanan S. (1997). Object detection using gabor filters. Pattern Recognit. 30, 295–309. 10.1016/S0031-3203(96)00068-4

[B38] Jing Y. Pavlović V. Rehg J. M. (2008). Boosted bayesian network classifiers. Mach. Learn. 73, 155–184. 10.1007/s10994-008-5065-7

[B39] Kime S. Galluppi F. Lagorce X. Benosman R. Lorenceau J. (2016). Psychophysical assessment of perceptual performance with varying display frame rates. J. Disp. Technol. 12, 1372–1382. 10.1109/JDT.2016.2603222

[B40] Kime S. Galluppi F. Lorenceau J. Benosman R. (2014). Exploring speed discrimination of visual stimuli at a high frame rate, in Annual Meeting of the Society For Neuroscience(SFN) (Washington, DC).

[B41] Lades M. Vorbruggen J. C. Buhmann J. Lange J. von der Malsburg C. Wurtz R. P. (1993). Distortion invariant object recognition in the dynamic link architecture. IEEE Trans. Comp. 42, 300–311.

[B42] Lagorce X. Benosman R. (2015). Stick: spike time interval computational kernel, a framework for general purpose computation using neurons, precise timing, delays, and synchrony. Neural Comput. 27, 2261–2317. 10.1162/NECO_a_00783 26378879

[B43] Lagorce X. Ieng S.-H. Benosman R. (2013). Event-based features for robotic vision, in IEEE/RSJ International Conference on Intelligent Robots and Systems (IROS) (Tokyo: IEEE), 4214–4219.

[B44] Lagorce X. Meyer C. Ieng S.-H. Filliat D. Benosman R. (2014). Asynchronous event-based multikernel algorithm for high-speed visual features tracking. Trans. Neural Netw. Learn. Syst. 26, 1710–1720. 10.1109/TNNLS.2014.2352401 25248193

[B45] Lagorce X. Orchard G. Galluppi F. Shi B. Benosman R (2016). Hots: a hierarchy of event-based time-surfaces for pattern recognition. IEEE Trans. Pattern Anal. Mach. Intell. Available online at: http://ieeexplore.ieee.org/abstract/document/7508476/ 10.1109/TPAMI.2016.2574707 27411216

[B46] Laptev I. (2005). On space-time interest points. Int. J. Comput. Vis. 64, 107–123. 10.1109/ICCV.2003.1238378

[B47] Lasserre J. A. Bishop C. M. Minka T. P. (2006). Principled hybrids of generative and discriminative models, in 2006 IEEE Computer Society Conference on Computer Vision and Pattern Recognition (CVPR'06), Vol. 1 (New York, NY: IEEE), 87–94.

[B48] Lee J. H. Delbrück T. Pfeiffer M. (2016). Training deep spiking neural networks using backpropagation. Front. Neurosci. 10:508. 10.3389/fnins.2016.00508 27877107 PMC5099523

[B49] Lee J. H. Delbrück T. Pfeiffer M. Park P. K. Shin C.-W. Ryu H. E.. (2014). Real-time gesture interface based on event-driven processing from stereo silicon retinas. IEEE Trans. Neural Netw. Learn. Syst. 25, 2250–2263. 10.1109/TNNLS.2014.2308551 25420246

[B50] Lichtsteiner P. Posch C. Delbrück T. (2008). A 128^*^128 120dB 15us latency asynchronous temporal contrast vision sensor. IEEE J. Solid State Circ. 43, 566–576. 10.1109/JSSC.2007.914337

[B51] Lyons M. Akamatsu S. Kamachi M. Gyoba J. (1998). Coding facial expressions with gabor wavelets, in Proceedings of Third IEEE International Conference on Automatic Face and Gesture Recognition (Nara: IEEE), 200–205.

[B52] Mikolajczyk K. Schmid C. (2005). A performance evaluation of local descriptors. IEEE Trans. Pattern Anal. Mach. Intell. 27, 1615–1630. 10.1109/TPAMI.2005.188 16237996

[B53] Milde M. Bertrand O. J. N. Benosman R. Egelhaaf M. Chicca E. (2015). Bioinspired event-driven collision avoidance algorithm based on optic flow, in Event-Based Control, Communication, and Signal Processing (EBCCSP) (Krakow).

[B54] Moeslund T. B. Hilton A. Kruger V. (2006). A survey of advances in vision-based human motion capture and analysis. Comput. Vis. Image Underst. 104, 90–126. 10.1016/j.cviu.2006.08.002

[B55] Mokhtarian F. Mohanna F. (2006). Performance evaluation of corner detectors using consistency and accuracy measureness. Comput. Vis. Image Understand. 102, 81–94. 10.1016/j.cviu.2005.11.001

[B56] Mokhtarian F. Suomela R. (1998). Robust image corner detection through curvature scale space. IEEE Trans. Pattern Anal. Mach. Intell. 20, 1376–1381.

[B57] Moravec H. (1980). Obstacle Avoidance and Navigation in the Real World by a Seeing Robot Rover. Technical report, CMU-RI-TR-80-03, Robotics Institute, Carnegie Mellon University and doctoral dissertation, Stanford University.

[B58] Moreels P. Perona P. (2007). Evaluation of features detectors and descriptors based on 3d objects. Int. J. Comput. Vis. 73, 263–284. 10.1007/s11263-006-9967-1

[B59] Mueggler E. Baumli N. Fontana F. Scaramuzza D. (2015a). Towards evasive maneuvers with quadrotors using dynamic vision sensors, in European Conference on Mobile Robots (ECMR) (Paris: IEEE), 1–8.

[B60] Mueggler E. Forster C. Baumli N. Gallego G. Scaramuzza D. (2015b). Lifetime estimation of events from dynamic vision sensors, in 2015 IEEE International Conference on Robotics and Automation (ICRA) (Seattle, WA: IEEE), 4874–4881.

[B61] Negri P. Clady X. Hanif S. M. Prevost L. (2008). A cascade of boosted generative and discriminative classifiers for vehicle detection. EURASIP J. Adv. Signal Process. 2008:136 10.1155/2008/782432

[B62] Ni Z. Ieng S.-H. Posch C. Régnier S. Benosman R. (2015). Visual tracking using neuromorphic asynchronous event-based cameras. Neural Comput. 20, 1–29. 10.1162/NECO_a_00720 25710087

[B63] Ni Z. Pacoret C. Benosman R. Régnier S. (2014). Haptic Feedback Teleoperation of Optical Tweezers. John Wiley and Sons.

[B64] Noble J. (1988). Finding corners. Image Vis. Comput. 6, 121–128.

[B65] Orban G. A. Wolf J. d. Maes H. (1984). Factors influencing velocity coding in the human visual system. Vis. Res. 24, 33–39. 6695505 10.1016/0042-6989(84)90141-x

[B66] Orchard G. Etienne-Cummings R. (2014). Bioinspired visual motion estimation. Proc. IEEE 102, 1520–1536. 10.1109/JPROC.2014.2346763

[B67] Orchard G. Lagorce X. Posch C. Furber S. B. Benosman R. Galluppi F. (2015a). Real-time event-driven spiking neural network object recognition on the spinnaker platform, in IEEE International Symposium on Circuits and Systems (ISCAS) (Lisbon: IEEE), 2413–2416.

[B68] Orchard G. Meyer C. Etienne-Cummings R. Posch C. Thakor N. Benosman R. (2015b). Hfirst: a temporal approach to object recognition. IEEE Trans. Pattern Anal. Mach. Intell. 37, 2028–2040. 10.1109/TPAMI.2015.2392947 26353184

[B69] Panaïté J. Usciati T. Clady X. Haliyo S. (2011). An experimental study of the kinect's depth sensor, in IEEE International Symposium on Robotic and Sensors Environment (Montreal).

[B70] Park S. Ahmad M. Seung-Hak R. Han S. Park J. (2004). Image corner detection using radon transform, in Computational Science and Its Applications, Vol. 3046, Lecture Notes in Computer Science, eds Lagano A. Gavrilova M. Kumar V. Mun Y. Tan C. Gervasi O. (Berlin; Heidelberg: Springer), 948–955.

[B71] Pérez-Carrasco J. A. Zhao B. Serrano C. Acha B. Serrano-Gotarredona T. Chen S.. (2013). Mapping from frame-driven to frame-free event-driven vision systems by low-rate rate coding and coincidence processing - application to feedforward convnets. IEEE Trans. Pattern Anal. Mach. Intell. 35, 2706–2719. 10.1109/TPAMI.2013.71 24051730

[B72] Poppe R. (2007). Vision-based Human motion analysis: an overview. Comput. Vis. Image Underst. 108, 4–18. 10.1016/j.cviu.2006.10.016

[B73] Poppe R. (2010). A survey on vision-based human action recognition. Image Vis. Comput. 28, 976–990. 10.1016/j.imavis.2009.11.014

[B74] Posch C. (2015). Bioinspired vision sensing, Biologically Inspired Computer Vision: Fundamentals and Applications, eds Cristóbal G. Perrinet L. Keil M. S. (Weinheim: Wiley-VCH Verlag GmbH & Co.). 10.1002/9783527680863.ch2

[B75] Posch C. Matolin D. Wohlgenannt R. (2010). High-DR frame-free PWM imaging with asynchronous AER intensity encoding and focal-plane temporal redundancy suppression, in Proceedings of 2010 IEEE International Symposium on Circuits and Systems (ISCAS) (Paris), 2430–2433.

[B76] Prevost L. Oudot L. Moises A. Michel-Sendis C. Milgram M. (2005). Hybrid generative/discriminative classifier for unconstrained character recognition. Pattern Recognit. Lett. 26, 1840–1848. 10.1016/j.patrec.2005.03.005

[B77] Priebe N. J. Lisberger S. G. Movshon J. A. (2006). Tuning for spatiotemporal frequency and speed in directionally selective neurons of macaque striate cortex. J. Neurosci. 26, 2941–2950. 10.1523/JNEUROSCI.3936-05.2006 16540571 PMC2532672

[B78] Rogister P. Benosman R. Ieng S.-H. Lichtsteiner P. Delbrück T. (2012). Asynchronous event-based binocular stereo matching. IEEE Trans. Neural Netw. Learn. Syst. 23, 347–353. 10.1109/TNNLS.2011.2180025 24808513

[B79] Rosten E. Drummond T. (2006). Machine learning for high-speed corner detection, in European Conference on Computer Vision (Graz), Vol. 1, 430–443.

[B80] Schapire R. E. Freund Y. Bartlett P. Lee W. S. (1998). Boosting the margin: A new explanation for the effectiveness of voting methods. Ann. Stat. 26, 1651–1686.

[B81] Schraudolph N. N. (1999). A fast, compact approximation of the exponential function. Neural Comput. 11, 853–862. 10226185 10.1162/089976699300016467

[B82] Schreiber S. Fellous J. M. Whitmer D. Tiesinga P. Sejnowski T. J. (2003). A new correlation based measure of spike timing reliability. Neurocomputing 52, 925–931. 10.1016/S0925-2312(02)00838-X 20740049 PMC2926980

[B83] Serrano-Gotarredona T. Linares-Barranco B. (2013). A 128x128 1.5% 20 contrast sensitivity 0.9% 20 fpn 3 μs latency 4 mw asynchronous frame-free dynamic vision sensor using transimpedance preamplifiers. IEEE J. Solid State Circ. 48, 827–838.

[B84] Simon-Chane C. Ieng S.-H. Posch C. Benosman R. B. (2016). Event-based tone mapping for asynchronous time-based image sensor. Front. Neurosci. 10:391. 10.3389/fnins.2016.00391 27642275 PMC5015463

[B85] Thrun S. Burgard W. Fox D. (2008). Probabilistic Robotics. MIT Press.

[B86] Valeiras D. R. Lagorce X. Clady X. Bartolozzi C. Ieng S.-H. Benosman R. (2015). An asynchronous neuromorphic event-driven visual part-based shape tracking. Trans. Neural Netw. Learn. Syst. 26, 3045–3059. 10.1109/TNNLS.2015.2401834 25794399

[B87] van Rossum M. (2001). A novel spike distance. Neural Comput. 13, 751–763. 10.1162/089976601300014321 11255567

[B88] Wang J. Xiao J. Lin W. Luo C. (2015). Discriminative and generative vocabulary tree: with application to vein image authentication and recognition. Image Vis. Comput. 34, 51–62. 10.1016/j.imavis.2014.10.014

[B89] Wang X. Clady X. Granata C. (2011). A human detection system for proxemics interaction, in Proceedings of the 6th International Conference on Human-Robot Interaction (Lausanne: ACM), 285–286.

[B90] Zhou H. Hu H. (2008). Human motion tracking for rehabilitation's survey. Biomed. Signal Process. Control 3, 1–18. 10.1016/j.bspc.2007.09.001

